# Multi-Parametric MRI-Based Radiomics Models for Predicting Molecular Subtype and Androgen Receptor Expression in Breast Cancer

**DOI:** 10.3389/fonc.2021.706733

**Published:** 2021-08-18

**Authors:** Yuhong Huang, Lihong Wei, Yalan Hu, Nan Shao, Yingyu Lin, Shaofu He, Huijuan Shi, Xiaoling Zhang, Ying Lin

**Affiliations:** ^1^Breast Disease Center, The First Affiliated Hospital, Sun Yat-sen University, Guangzhou, China; ^2^Department of Pathology, The First Affiliated Hospital, Sun Yat-sen University, Guangzhou, China; ^3^Department of Radiology, The First Affiliated Hospital, Sun Yat-sen University, Guangzhou, China

**Keywords:** breast cancer, radiomics, molecular subtype, androgen receptor, magnetic resonance imaging, machine learning

## Abstract

**Objective:**

To investigate whether radiomics features extracted from multi-parametric MRI combining machine learning approach can predict molecular subtype and androgen receptor (AR) expression of breast cancer in a non-invasive way.

**Materials and Methods:**

Patients diagnosed with clinical T2–4 stage breast cancer from March 2016 to July 2020 were retrospectively enrolled. The molecular subtypes and AR expression in pre-treatment biopsy specimens were assessed. A total of 4,198 radiomics features were extracted from the pre-biopsy multi-parametric MRI (including dynamic contrast-enhancement T1-weighted images, fat-suppressed T2-weighted images, and apparent diffusion coefficient map) of each patient. We applied several feature selection strategies including the least absolute shrinkage and selection operator (LASSO), and recursive feature elimination (RFE), the maximum relevance minimum redundancy (mRMR), Boruta and Pearson correlation analysis, to select the most optimal features. We then built 120 diagnostic models using distinct classification algorithms and feature sets divided by MRI sequences and selection strategies to predict molecular subtype and AR expression of breast cancer in the testing dataset of leave-one-out cross-validation (LOOCV). The performances of binary classification models were assessed *via* the area under the receiver operating characteristic curve (AUC), accuracy, sensitivity, specificity, positive predictive value (PPV), and negative predictive value (NPV). And the performances of multiclass classification models were assessed *via* AUC, overall accuracy, precision, recall rate, and F1-score.

**Results:**

A total of 162 patients (mean age, 46.91 ± 10.08 years) were enrolled in this study; 30 were low-AR expression and 132 were high-AR expression. HR+/HER2− cancers were diagnosed in 56 cases (34.6%), HER2+ cancers in 81 cases (50.0%), and TNBC in 25 patients (15.4%). There was no significant difference in clinicopathologic characteristics between low-AR and high-AR groups (P > 0.05), except the menopausal status, ER, PR, HER2, and Ki-67 index (P = 0.043, <0.001, <0.001, 0.015, and 0.006, respectively). No significant difference in clinicopathologic characteristics was observed among three molecular subtypes except the AR status and Ki-67 (P = <0.001 and 0.012, respectively). The Multilayer Perceptron (MLP) showed the best performance in discriminating AR expression, with an AUC of 0.907 and an accuracy of 85.8% in the testing dataset. The highest performances were obtained for discriminating TNBC *vs.* non-TNBC (AUC: 0.965, accuracy: 92.6%), HER2+ *vs.* HER2− (AUC: 0.840, accuracy: 79.0%), and HR+/HER2− *vs.* others (AUC: 0.860, accuracy: 82.1%) using MLP as well. The micro-AUC of MLP multiclass classification model was 0.896, and the overall accuracy was 0.735.

**Conclusions:**

Multi-parametric MRI-based radiomics combining with machine learning approaches provide a promising method to predict the molecular subtype and AR expression of breast cancer non-invasively.

## Introduction

According to the International Agency for Research on Cancer, breast cancer has become the most prevalent cancer and the leading cause of cancer death in women worldwide (1). Breast cancer is a highly heterogeneous disease and can be classified into different molecular subtypes based on the expression of several specific molecular receptors. The biological diversity of breast cancer is associated with various clinical manifestations, treatment responses, and prognoses (2). Based on the expression of estrogen receptor (ER), progesterone receptor (PR), and human epidermal growth factor receptor-2 (HER2), breast cancers are classified into three distinct molecular subtypes as follows: hormone receptor (HR) positive and HER2 negative (HR+/HER2−), HER2+, and triple-negative breast cancer (TNBC) (3). This classification system is widely used to guide individual systematic therapy of breast cancer: HR+ patients require effective endocrine therapy; HER2+ patients require anti-HER2 therapy; TNBC patients require cytotoxic therapy. Thus, it is crucial to detect the ER, PR, and HER2 status to select the optimal treatment for patients with breast cancer. However, the current treatment strategies have some limitations. It has been reported that 30–50% of patients with ER+ tumors were resistant to endocrine treatment (4). Approximately 65% of patients with HR−/HER2+ tumors did not respond to anti-HER2 treatment, and about 70% of patients who initially responded experienced tumor progression after treatment (5). TNBC is considered the most aggressive phenotype of breast cancer with a very poor prognosis due to the absence of specific targeted treatment. Consequently, there has been increased foci on developing novel biomarkers to guide clinical decisions for breast cancer (6–13).

The androgen receptor (AR) has been reported as a prognostic biomarker which provides additional information and might be a viable therapeutic target in breast cancer (10–12, 14–18). AR is a member of the steroid hormone receptor superfamily of ligand-activated transcription factors and is overexpressed in 70–90% of breast cancers (10, 14). Furthermore, AR expression is variable in different subtypes of breast cancer, and high AR expression was reported in about 75% of HR+ cases, 50–60% of HER2+ cases, and 20–40% of TNBC cases, respectively (19). According to some current studies, AR has been confirmed as a biomarker associated with a favorable prognosis of breast cancer in terms of disease-free survival (DFS) and overall survival (OS) (15, 20, 21). In patients with HER2+ breast cancer who underwent neoadjuvant chemotherapy, high AR expression was associated with a better therapeutic response (11). Although TNBCs are frequently grouped together due to lack of ER, PR, and HER2 expression, TNBCs actually were heterogeneous. Among the six molecular subtypes of TNBCs proposed by Lehmann et al., the luminal androgen receptor subtype, characterized by the AR expression, was associated with an improved prognosis compared to other subtypes (10, 22, 23). Additionally, AR as a therapeutic target is under the clinical investigation for breast cancer, especially in the TNBCs or tumors resistant to first-line targeted therapy (18, 24, 25).

Thus, detecting molecular subtype and AR expression of breast cancer is important for treatment selection and predicting therapeutic response. For tumor larger than 2 cm (clinical T2–4 stage) with/without axillary metastasis, if the molecular subtype and AR expression could be identified before surgery, we can determine whether the patient is suitable for neoadjuvant chemotherapy and which scheme should be used. However, currently, we cannot obtain histologic information by routine imaging examinations including mammography, ultrasound, and magnetic resonance imaging (MRI). Breast biopsy is still the standard operation to acquire histologic characteristics of breast cancer. However, breast biopsy is an invasive surgical procedure and is inapplicable for some patients. Besides, the assessment of molecular subtype and AR expression from pre-treatment biopsy specimen cannot reflect the change of receptors expression during treatment. Thus, an alternative method that can accurately and non-invasively evaluate the expression of receptors in breast cancer is an urgent requirement, which would be helpful to guide clinical treatment.

In recent years, an increasing interest has been focused on identifying imaging surrogates and developing non-invasive diagnostic tools for cancer characterization (26). Due to rapid advancements in quantitative radiology methods such as radiomics, radiogenetics, and radioproteomics, tumor biology could be evaluated in a non-invasive and cost-effective way. Radiomics allows inference of tumoral molecular status from medical image-derived features, and it allows the study of the tumoral heterogeneity both spatially and over time (27, 28). Multi-parametric MRI, including dynamic contrast-enhancement (DCE) images, diffusion-weighted images (DWI), and other MRI sequences, is a well-established imaging modality for diagnosis, pre-operative staging, and surgical planning of breast cancer in current routine clinical practice. DCE imaging is considered the most sensitive modality for detecting breast cancer (29). Ruey-Feng Chang et al. reported that the degree of heterogeneity on breast DCE-MRI was associated with molecular receptor status (30). DWI and its derived apparent diffusion coefficient (ADC) map also served as a non-contrast MR screening method in lesion detection and distinguishing malignancy from benign tumor (31). Several studies applied radiomics based on breast MRI for the evaluation of malignancy, differentiation of molecular subtype, prediction of receptor expression, and evaluation of response to neoadjuvant therapy in breast cancer (27, 32–35). Some studies have reported that quantitative parameters of functional MRI, deep learning analysis, and MRI-based radiomics analysis had the potential in predicting molecular subtype and Ki-67 expression in breast cancer (36–45). However, no published study reported the accuracy of MRI combining with radiomics in predicting AR expression and explored the importance of different MRI sequences in predicting molecular subtype of breast cancer. The feasibility of multi-parametric MRI-based radiomics models in predicting molecular subtype and AR expression of breast cancer still needs to be explored. Compared to previous studies, we firstly investigated the further radiomics analysis using multi-parametric MRI including DCE, T2-weighted images (T2WI), and ADC images for the breast cancer molecular subtype classification and AR expression. To analyze which type of feature and machine learning method will affect the classifier, we compared the performance of radiomics features from different types of MR image and their combinations, and we also compared the performance of three different feature selection strategies and several supervised classification algorithms. In our study, to explore the correlations between the receptor expression and MRI-derived radiomics features, we used partial dependency plot (PDP) to explain radiomics feature, which was a supplement to other published studies.

## Materials and Methods

### Study Population

This study was approved by the Ethical Committee of the First Affiliated Hospital, Sun Yat-sen University, and the requirement for written informed consent was waived due to the retrospective nature of this study. We retrieved 1,947 consecutive patients with breast cancer who underwent breast multi-parametric MRI examination and following treatment in our center from March 2016 to July 2020. The inclusion criteria were as follows: (I) the patient had a histologically proven invasive breast cancer and a histologic result of AR, ER, PR, HER2, and Ki-67 expression; (II) the breast MRI examination was performed before biopsy or anti-tumor treatment within 1 month; (III) the tumor was not smaller than 2 cm (clinical T2–4 stage), in order to ensure sufficient information of MRI-derived radiomics features and reduce the influence of partial volume effect; (IV) the patient had complete baseline data. The exclusion criteria were as follows: (I) lack of MRI examination or poor imaging quality; (II) the patient had bilateral breast carcinoma or multifocal lesions in the ipsilateral breast; (III) the patient had known distant metastasis or another malignancy; (IV) lack of histologically proven receptor status. Finally, a total of 162 patients (all were females; mean age: 46.9 ± 10.08 years, range: 23–78 years) with 162 invasive breast cancers (clinical T2–4 stage) were enrolled in this study.

### MRI Protocol

All the breast MRI examinations were performed using a 3.0 Tesla (T) MR scanner (Verio, Siemens Healthcare, Erlangen, Germany) with a dedicated 16-channel phased-array breast coil. During the MRI examination, the breasts of the patient were fixed with the prone position. The multi-parametric MRI sequences included a transverse T1-weight dynamic contrast-enhancement image (T1-DCE), a transverse fat-suppressed T2-weighted image (FS-T2WI), a transverse DWI, and a DWI-derived ADC map.

All the MRI sequences used for analysis in our study met the standard MR imaging acquisition procedure. Firstly, a transverse FS-T2WI was obtained. Secondly, a transverse DWI was performed using an echo-planar imaging sequence. Thirdly, a transverse T1-weighted image was acquired immediately before contrast agent injection and at six consecutive time points after the contrast agent injection. The contrast agent injection was as follows: After finishing the conventional MRI sequences acquisition, the gadolinium contrast agent (Magnevist, Bayer HealthCare Pharmaceuticals Inc., Wayne, USA) was intravenously injected at a dose of 0.1 mmol per kilogram of body weight and a rate of 2 ml/s. When the contrast agent injection was over, a 20 ml saline flush was followed. The ADC value was calculated in the MRI workstation as follow:

ADC value=[lnS0−lnS(b)]/b

where S0 is the DWI signal intensity at b = 0 s/mm^2^, S(b) is the DWI signal intensity at b = 1,000 s/mm^2^. The detailed MRI scanning parameters of T1-DCE, FS-T2WI, and ADC map are listed in **Table 1**.

**Table 1:** Scanning parameters for three MRI sequences.

Scanning parameter	TR/TE	PD	FOV	MS	SL	SR	ST
T1-DCE	4.32/1.57	446	380×380	448×448	144	0.848×0.848	1.0
FS-T2WI	4330/61	319	380×380	320×320	38-42	1.188×1.188	3.0
ADC map	6300/74	2083	380×380	160×160	24-32	2.375×2.375	4.0

TR/TE, repeat time/echo time (ms); PD, Pixel bandwidth; FOV, field of view (mm); MS, Matrix size; SL, Slicer layer; SR, Spatial resolution (mm^2^); ST, Slice thickness (mm); T1-DCE, T1-weighted dynamic contrast-enhancement imaging; FS-T2WI, Fat suppression T2-weighted imaging; ADC map, apparent diffusion coefficient map.

### Pathological Analysis

A professional breast surgeon performed the ultrasound-guided breast biopsy using a 14G core needle, and more than three tumor tissue samples were acquired per patient. Then the samples were fixed by formalin and embedded by paraffin, and stained by hematoxylin and eosin (H&E staining). Immunohistochemical (IHC) analysis was used to determine the ER, PR, HER2, Ki-67, and AR expression.

The ER and PR status were defined as positive if ≥1% of tumor cells showing positively stained nuclei. For HER2 status determination, an IHC score 3+ was defined as positive, while an IHC score 0 or 1+ was defined as negative. An IHC score 2+ of HER2 was considered indeterminate. Fluorescence *in situ* hybridization (FISH) was performed to assess gene amplification, and HER2 was considered positive if the ratio ≥2.0. Ki-67 expression was evaluated by calculating the percentage of tumor cells with positively stained nuclei from at least 500 cells in a slicer. We set 30% as the cut-off value, and <30% was considered Ki-67 low expression, while ≥30% was considered Ki-67 high expression. According to the IHC and FISH results, breast cancers were classified into three subtypes in our study: (I) HR+/HER2− (ER+ and/or PR+, and HER2−); (II) HER2+ (ER−, PR−, and HER2+); (III) TNBC (ER−, PR−, and HER2−). The AR status was defined as positive if ≥10% of tumor cells showed positive staining.

### Image Processing, ROI Delineation, and Radiomics Analysis

The image processing, delineation of tumor region of interest (ROI), and radiomics analysis contained three steps: (1) imaging preprocessing to all the MRI sequences; (2) segmentation of the ROIs; (3) extraction of radiomics feature. The bias field of MRI scanning could cause variation in imaging signal intensity, which was not caused by any biological differences of breast cancer. We used N4 Bias Field Correction package to correct the bias field before tumor segmentation. Image normalization was necessary for all MRI sequences to achieve intensity homogeneity, so the range of voxel intensity in MR image was scaled to 0–2,000 to avoid the influence of imaging intensity inconsistency.

After imaging preprocessing, two radiologists with ≥10 years of clinical experience in breast MRI drew the ROIs of the breast cancers in T1-DCE due to the high intensity of tumor in this sequence. When there was a disagreement about the tumor margin, an elder radiologist with 22 years of clinical experience in breast MRI made the final decision to the ROI after carefully distinguishing the tumor region. Then the ROIs in T1-DCE were then registered and applied to the other two sequences (FS-T2WI and ADC map), and the MRI slicers and orientation were matched carefully between T1-DCE and FS-T2WI (or ADC map). During ROI segmentation, the necrosis, air, and calcification area of the breast cancer were excluded carefully. Finally, we completed ROI delineation in all the MRI sequences.

Radiomics features were extracted using Pyradiomics package plugged in the 3D-slicer software. Before feature extraction, spacing standardization of MR images was done to ensure a uniform voxel spacing (1.0 × 1.0 × 1.0 mm^3^) in the three-dimensional space. A total of 4,230 radiomics features were extracted from ROI in three sequences (1410 features for each sequence). For each MRI sequence, 19 intensity-based first-order statistical features, 17 shape-based features (3D), 24 gray level co-occurrence matrix (GLCM) features, 16 gray level size zone matrix (GLSZM) features, 16 gray level run length matrix (GLRLM) features, 5 neighborhood gray-tone difference matrix (NGTDM) features, and 14 neighboring gray level dependence matrix (NGLDM) features were extracted from the original images. Moreover, we used Laplacian of Gaussian imaging filters (kernel size: 1, 2, 3, 4, 5, and 6) and wavelet imaging filters to deal with all the original images and generate more images, and a total of 1299 intensity-based first-order statistical features and texture features were then calculated and extracted from those derived images. Removing the redundant shape-based features (16 features extracted from FS-T2WI and ADC map, respectively), a total of 4,198 radiomics features were extracted and used for the following analysis per patient. The details of radiomics features calculation formulas are listed in **Supplementary Material**.

### Feature Selection

Inter- and intra-class correlation coefficients (ICCs) were calculated to evaluate the inter-observer and intra-observer reproducibility of all the radiomics features. Thirty cases of MRI containing 15 AR− and 15 AR+ were randomly chosen. For the intra-class ICCs, the ROI segmentation was done by two radiologists skilled in breast MRI independently. For the inter-class ICCs, radiologist 1 repeated the segmentation work 1 month after the completion of ROI segmentation of these cases. Radiomics features with inter- and intra-class ICCs >0.75 were considered having good reproducibility and could be selected for model construction.

Some features might improve the performance of classification model, while others might reduce that, so it is necessary to choose the meaningful features relevant to the AR expression and molecular subtype. For dimensionality reduction of the total radiomics features, we used three feature selection strategies to select the optimal features as follows: (1) the least absolute shrinkage and selection operator (LASSO) and following recursive feature elimination (RFE) method; (2) the maximum relevance–minimum redundancy (mRMR) method (46); (3) the Boruta method (47). For LASSO and RFE algorithm, 10-fold cross-validation was used to ensure the robustness of the selected features. Firstly, the dimension of features was reduced to 100 (we considered that the 100 features is enough to cover the most valuable features as LASSO is suitable to process high-dimensional and small-sample size data with the collinearity) by LASSO. Then the most significant features were further identified by RFE among these 100 features. The number of retained features was determined according to the best average accuracy in the testing dataset with a robust Random Forest (RF) classifier. For mRMR algorithm, it was used to select the features that are most relevant to the predictive labels and eliminate the redundant feature. We obtained the top 30 features and then evaluated the inter-class distribution difference and AUC value of each feature. Only the features with significant distribution difference (p-value <0.05) or an AUC value that >0.5 were selected (for predicting molecular subtypes, feature with a significant distribution difference between any two subtypes was retained). For Boruta algorithm, the importance of each feature was calculated by 100 times, and an average importance value was obtained, then the features with an average importance value higher than that of the shadow feature were remained. The details of these three feature selection methods are described in **Supplementary Material**. Finally, a pairwise Pearson correlation coefficient matrix (PCCM) was then used to identify any pair of features with high correlation. If the absolute value of correlation coefficient ≥0.8, a high correlation between two features was considered, and only one feature with a higher performance would be remained.

### Model Development and Evaluation

To maximize the utilization of samples and ensure the robustness of models, we used the leave-one-out cross-validation (LOOCV) method to construct machine learning models. All patients with breast cancer were divided into the training dataset (161 patients) and the testing dataset (the rest one patient) in turn, and 162 times of data splitting were performed. In each loop of LOOCV, the total radiomics features extracted from three MRI sequences were used. In the training dataset split by LOOCV, to ensure the uniform scale of feature value, all the radiomics features were standardized using z-score normalization, and the following is the calculating formula:

y=(x−μ)/σ

where *x* is the original value of feature, and *μ* and *σ* are the mean and standard deviation values of *x*, respectively, and *y* is the transformed feature value. Then the features of patient in the testing dataset were transformed according to the corresponding feature value in the training dataset.

We retained the features selected from three feature selection methods for predicting molecular subtype and AR expression, respectively. Finally, the 23 features (10 features from DCE-MRI, 9 features from T2WI, and 4 features from ADC-map) were retained for predicting AR expression, and 30 features (7 features from DCE-MRI, 14 features from T2WI, and 9 features from ADC-map) were retained for predicting molecular subtypes. And to evaluate which type of features divided by MRI sequences or selection strategies would influence the model performance, we compared the model performances developed with different feature sets as follows: (1) total features, (2) features selected by LASSO-RFE, (3) features selected by mRMR, (4) features selected by Boruta, (5) features from DCE-MRI, (6) features from T2WI, (7) features from ADC-map, (8) features from DCE-MRI and T2WI, (9) features from DCE-MRI and ADC-map, and (10) features from T2WI and ADC-map. Those 10 feature sets were used to develop the prediction models.

For predicting AR expression, we applied six supervised classification algorithms, including RF, Logistic Regression (LR), Gaussian Naïve Bayes (GNB), Linear Discriminant Analysis (LDA), Support Vector Machine (SVM, based on radial basis function), and Multilayer Perceptron (MLP). For predicting molecular subtype, we applied six supervised classification algorithms, including RF (based on the “one *vs.* one” decision function), LR (based on the “one *vs.* rest” decision function), Gaussian process classifier (GPC, based on the “one *vs.* rest” decision function), SVM (based on radial basis function and the “one *vs.* one” decision function), Linear Discriminant Analysis (LDA), and Multilayer Perceptron (MLP). The details of machine learning algorithms are described in **Supplementary Material**. The hyper-parameters of above machine learning algorithms were tuned by grid search approach and 10-fold cross-validation in the training dataset. In each loop of LOOCV, the hyper-parameters with the best AUC in the validation dataset were retained and used for the final model establishment using the whole training dataset. And the rest one patient in the testing dataset was used to evaluate the model performance. After finishing one round of LOOCV, each patient would get a predicted probability of the corresponding predicted label. The details of tuning hyper-parameters of different machine learning algorithms are described in **Supplementary Materials**.

**Figure 1:**
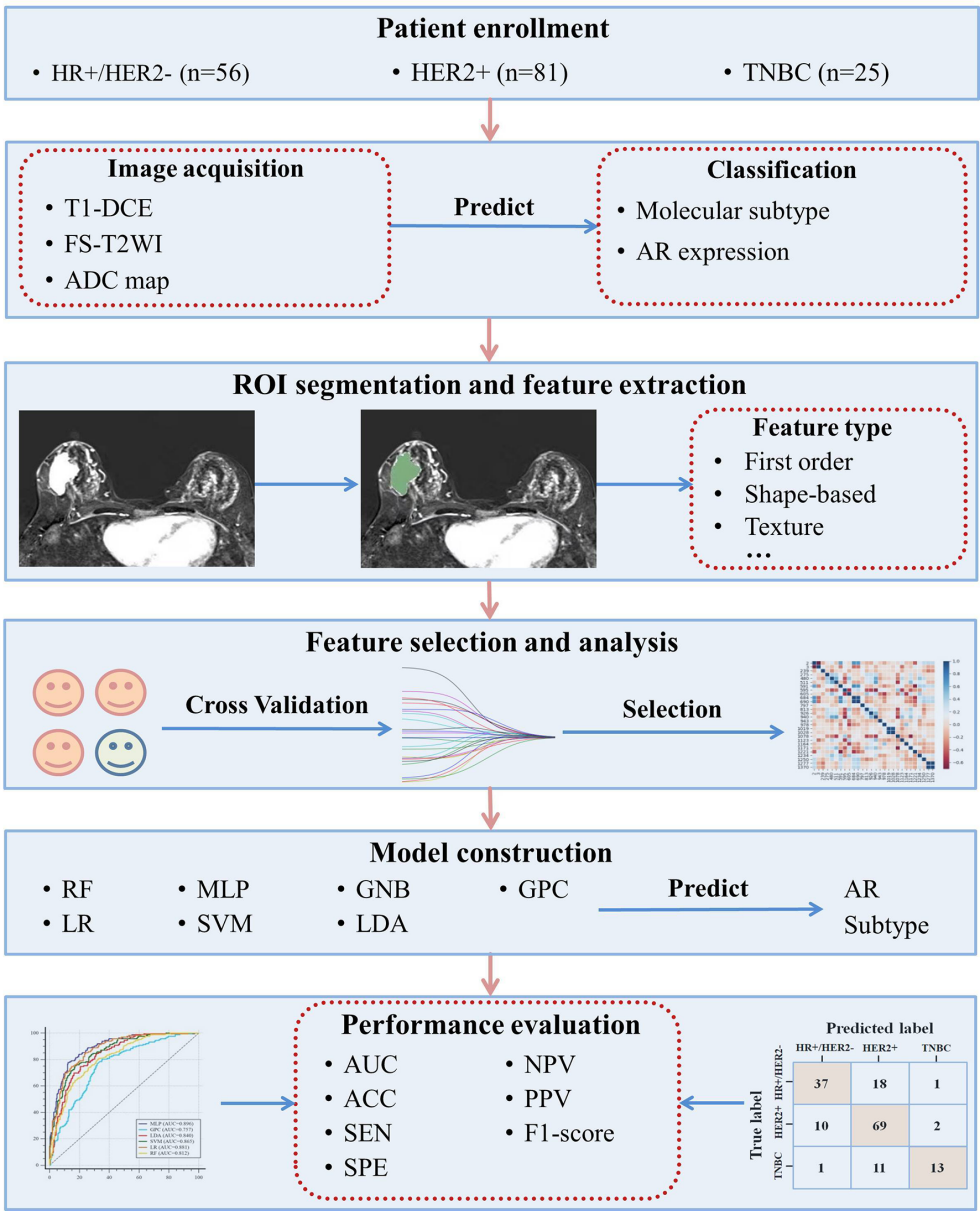
The workflow of this study.

To intuitively demonstrate the model performance of predicting AR expression, the receiver operating characteristic (ROC) curve was applied. The area under the curve (AUC) and other diagnostic indexes, including accuracy, sensitivity, specificity, positive predictive value (PPV), and negative predictive value (NPV), were used to evaluate the diagnostic performance of models in the testing dataset. While for evaluating the multiclass model performances of predicting molecular subtype, we used the micro-AUC and weighted macro-AUC to evaluate the comprehensive model performance, and the AUC in different subtypes were evaluated as well. The F1-score, precision, recall rate, and overall accuracy were also calculated based on different subtypes and models in the testing dataset. The workflow of this study is presented in **Figure 1**. Then, to evaluate the importance of those features contributing to the prediction results, the Shapley (SHAP) value of each feature was calculated in the model with the best AUC, and the importance rankings of features were shown as bar plots.

### Statistical Analysis

The data were calculated and analyzed using SPSS (software version, 22.0). All numeric data were calculated and expressed as the mean ± standard deviation (SD), while categorical data were expressed as the relative distribution frequency and percentage. The Kolmogorov-Smirnov test and F-test were used to evaluate the normality and homogeneity of variance of the numeric data, respectively. The independent t-test, Fisher’s exact test, and Mann-Whitney U test were applied to compare the baseline characteristics for numeric variables. The Chi-square test was applied for categorical variables between AR− and AR+ population cohorts and different subtypes. Pearson’s coefficients were calculated to analyze the relationship between the radiomics features and the baseline characteristics. The Mann-Whitney U test was used to compare the differences of the diagnostic performance (accuracy, sensitivity, specificity, PPV, NPV, precision, and recall rate) between the predictive models. The 95% confidence interval of AUC was calculated by the De-long test. P-value <0.05 was considered statistically significant.

## Results

### Clinicopathologic Characteristics

In total, 162 females with invasive breast cancer were identified and included in our study. The mean age of all the patients was 46.91 ± 10.08 years (age range, 23–78 years). Of the 162 patients, 30 patients (18.5%) had histologically confirmed low AR expression, while 132 patients (81.5%) had high AR expression. The distribution based on molecular subtype was as follows: 56 were HR+/HER2− (34.6%), 81 were HER2+ (50.0%), and 25 were TNBC (15.4%). There was no significant interclass difference in age, histologic tumor type, clinical anatomic stage, clinical T stage, and clinical N stage between the low-AR and high-AR expression groups (p-value = 0.335, 0.350, 0.377, 0.873, and 0.412, respectively). The characteristics with statistically significant differences were menopausal status, ER, PR, HER2, and Ki-67 expression between those two groups (p-value = 0.043, <0.001, <0.001, 0.015, and 0.006, respectively). Among the three molecular subtypes, the AR and Ki-67 expression showed significant differences (p-value = <0.001 and 0.012, respectively). No significant differences were found across other clinical characteristics among those three molecular subtypes. The clinical and histopathologic characteristics of patients are summarized in **Tables 2** and **3**.

**Table 2:** Clinical and histopathologic characteristics of patients grouped by AR expression.

Characteristics	AR <10% (n = 30)	AR ≥10% (n = 132)	p-value
Age (years)*	45.30 ± 11.04	47.27 ± 9.85	0.335
Menopausal Status			0.043
Premenopausal	10 (33.3%)	71 (53.8%)	
Postmenopausal	20 (66.7%)	61 (46.2%)	
Histologic type			0.353
IDC	30 (100.0%)	124 (93.9%)	
Other	0 (0.0%)	8 (6.1%)	
Clinical stage			0.377
II	13 (43.3%)	69 (52.3%)	
III	17 (56.7%)	63 (47.7%)	
Clinical T stage			0.873
cT2	20 (66.7%)	90 (68.2%)	
cT3-4	10 (33.3%)	42 (31.8%)	
Clinical N stage			0.412
cN0	5 (16.7%)	18 (13.6%)	
cN1	12 (40.0%)	70 (53.0%)	
cN2-3	13 (43.3%)	44 (33.3%)	
ER status			<0.001
Positive	8 (26.7%)	87 (65.9%)	
Negative	22 (73.3%)	45 (34.1%)	
PR status			<0.001
Positive	4 (13.3%)	79 (59.9%)	
Negative	26 (86.7%)	53 (40.2%)	
HER2 status			0.015
Positive	9 (30.0%)	72 (54.6%)	
Negative	21 (70.0%)	60 (45.5%)	
Molecular subtype			<0.001
HR+/HER2−	6 (20.0%)	50 (37.9%)	
HER2+	9 (30.0%)	72 (54.6%)	
TNBC	15 (50.0%)	10 (7.6%)	
Ki-67			0.006
<30%	6 (20.0%)	63 (47.7%)	
≥30%	24 (80.0%)	69 (52.3%)	

Data are described as numbers of patients, with percentages in the parentheses. *Those data are described as mean ± standard deviation. AR, androgen receptor; IDC, invasive ductal breast carcinoma; ER, estrogen receptor; PR, progesterone receptor; HER2, human epidermal growth factor receptor-2; Ki-67, cellular proliferation index; HR, hormone receptor; TNBC, triple-negative breast cancer.

**Table 3:** Clinical and histopathologic characteristics of patients grouped by molecular subtypes.

Characteristics	Total patients	Molecular subtypes			p-value
		HR+/HER2−	HER2+	TNBC	
Age (years)*	46.91 ± 10.08	46.43 ± 8.34	47.56 ± 10.78	45.88 ± 11.46	0.687
Menopausal Status					
Premenopausal	81 (50.0%)	31 (55.4%)	40 (49.4%)	10 (40.0%)	0.437
Postmenopausal	81 (50.0%)	25 (44.6%)	41 (50.6%)	15 (60.0%)	
Histologic type					
IDC	154 (95.1%)	51 (91.1%)	79 (97.5%)	24 (96.0%)	0.211
Other	8 (4.9%)	5 (8.9%)	2 (2.5%)	1 (4.0%)	
Clinical stage					
II	82 (50.6%)	26 (46.4%)	47 (58.0%)	9 (36.0%)	0.116
III	80 (49.4%)	30 (53.6%)	34 (42.0%)	16 (64.0%)	
Clinical T stage					
cT2	110 (67.9%)	39 (69.6%)	57 (70.4%)	14 (56.0%)	0.381
cT3-4	52 (32.1%)	17 (30.4%)	24 (29.6%)	11 (44.0%)	
Clinical N stage					
cN0	23 (14.2%)	5 (8.9%)	15 (18.5%)	3 (12.0%)	0.622
cN1	82 (50.6%)	29 (51.8%)	40 (49.4%)	13 (52.0%)	
cN2-3	57 (35.2%)	22 (39.3%)	26 (32.1%)	9 (36.0%)	
AR					
<10%	30 (18.5%)	6 (10.7%)	9 (11.1%)	15 (60.0%)	<0.001
≥10%	132 (81.5%)	50 (89.3%)	72 (88.9%)	10 (40.0%)	
Ki-67					
<30%	69 (42.6%)	28 (50.0%)	37 (45.7%)	4 (16.0%)	0.012
≥30%	93 (57.4%)	28 (50.0%)	44 (54.3%)	21 (84.0%)	

Data are described as numbers of patients, with percentages in the parentheses. *Those data are described as mean ± standard deviation. AR, androgen receptor; IDC, invasive ductal breast carcinoma; ER, estrogen receptor; PR, progesterone receptor; HER2, human epidermal growth factor receptor-2; Ki-67, cellular proliferation index; HR, hormone receptor; TNBC, triple-negative breast cancer.

### Feature Selection and Radiomics Component Development

**Figure 2:**
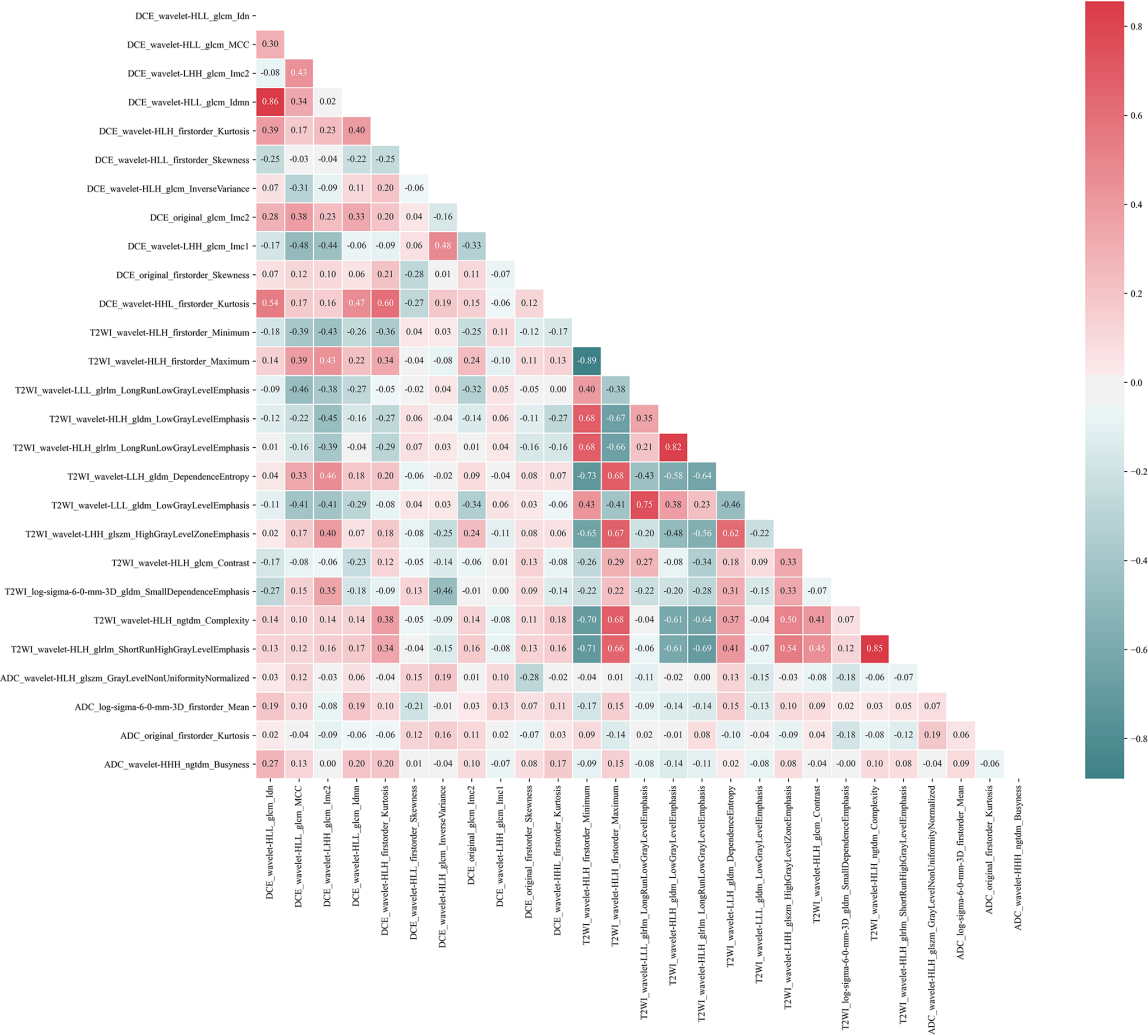
Pearson correlation coefficient heatmap of selected features on predicting AR expression. Red color denotes a positive correlation, and green color denotes a negative correlation, and the shade of the color indicates the correlation degree.

**Figure 3:**
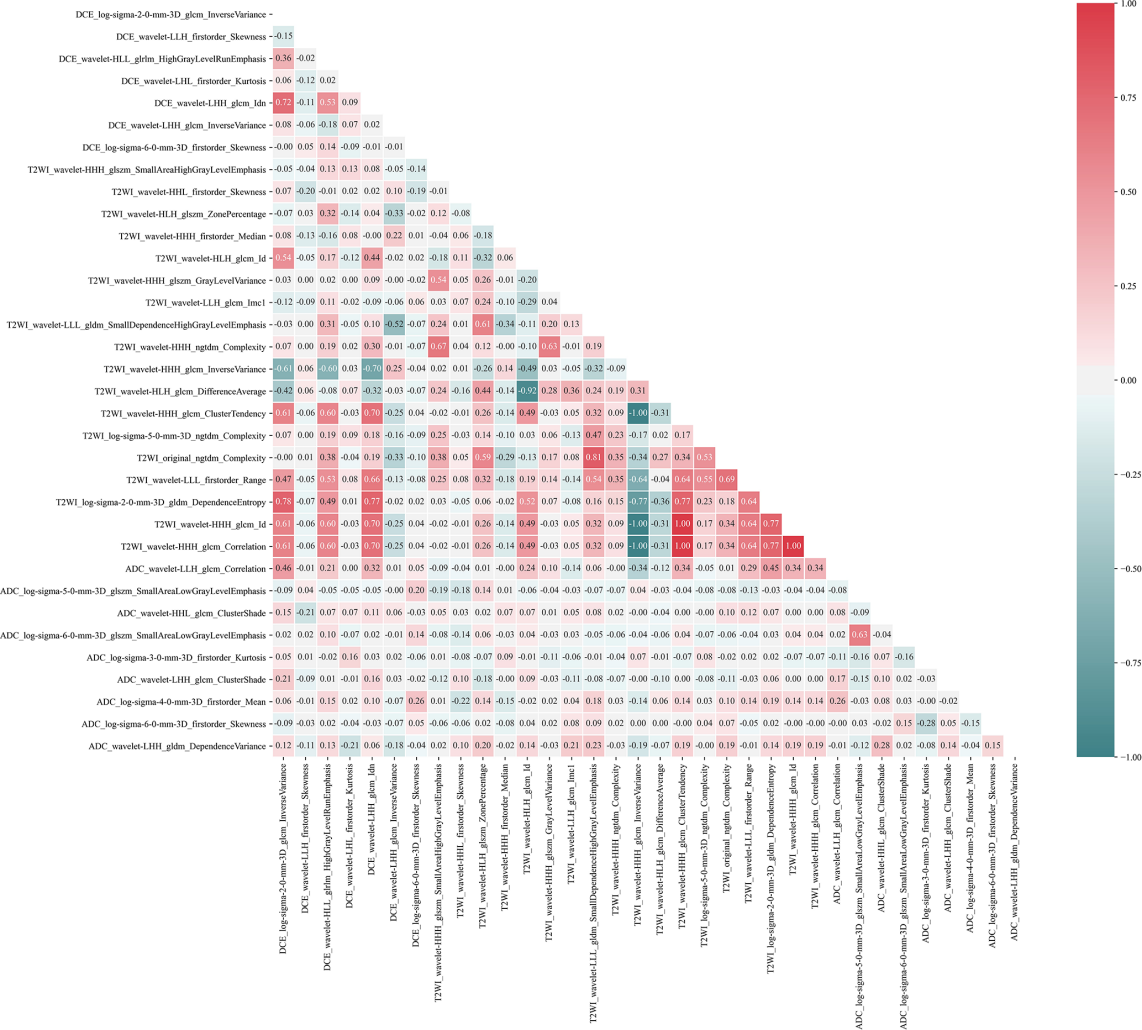
Pearson correlation coefficient heatmap of selected features on predicting molecular subtype. Red color denotes a positive correlation, and green color denotes a negative correlation, and the shade of the color indicates the correlation degree.

For radiomics features extracted from the T1-DCE, T2WI, and ADC-map, 1,368, 1,275, and 1,320 features showed reliable with an ICC higher than 0.75, respectively. Thus, a total of 3,963 features were used for further analysis. After LASSO-RFE fusion feature selection, the seven and the five most optimal radiomics features were selected for predicting molecular subtype and AR expression of breast cancer, respectively. After mRMR feature selection, 16 and 11 radiomics features were retained for predicting those two histologic outcomes, respectively. While after Boruta feature selection, 13 and 14 radiomics features were retained. Combining the total features selected from three strategies, we finally got 27 features and 34 features for predicting AR expression and molecular subtype of breast cancer, respectively. Then the Pearson correlation coefficient between any pair of these features was calculated, and the Pearson correlation coefficient matrix heatmaps are shown in **Figures 2** and **3**. There was multi-collinearity between some radiomics features retained in both feature sets. The Pearson correlation coefficient was in a range of −0.89–0.86 and −1.0–1.0 in two feature sets, respectively (if the absolute value of correlation coefficient ≥0.8, there was considered a high correlation between two features, and only one feature with a higher AUC or a significant inter-group distribution difference would be retained). After deleting the features with high correlation coefficient, 23 and 30 features were retained for predicting AR expression and molecular subtype, respectively.

The mean value and standard deviation of each feature were calculated to describe the inter-group distribution. The AUC was applied to assess the diagnostic performance of each radiomics feature for predicting AR expression. The mean value and standard deviation and the AUC values with 95% confidence intervals (only for binary classification model) of the selected features are listed in **Supplementary Materials**. For predicting molecular subtypes, we retained 23 features after feature selection as follows: 7 features from DCE-MRI, 14 features from T2WI, and 9 features from ADC-map. For predicting AR expression, we retained 30 features after feature selection as follows: 10 features from DCE-MRI, 9 features from T2WI, and 4 features from ADC-map. Each MRI sequence had retained radiomics features for subsequent analysis to predict molecular subtype and AR expression, so we considered using multi-parametric MRI was reasonable and valuable.

### Model Construction and Performance Evaluation

**Figure 4:**
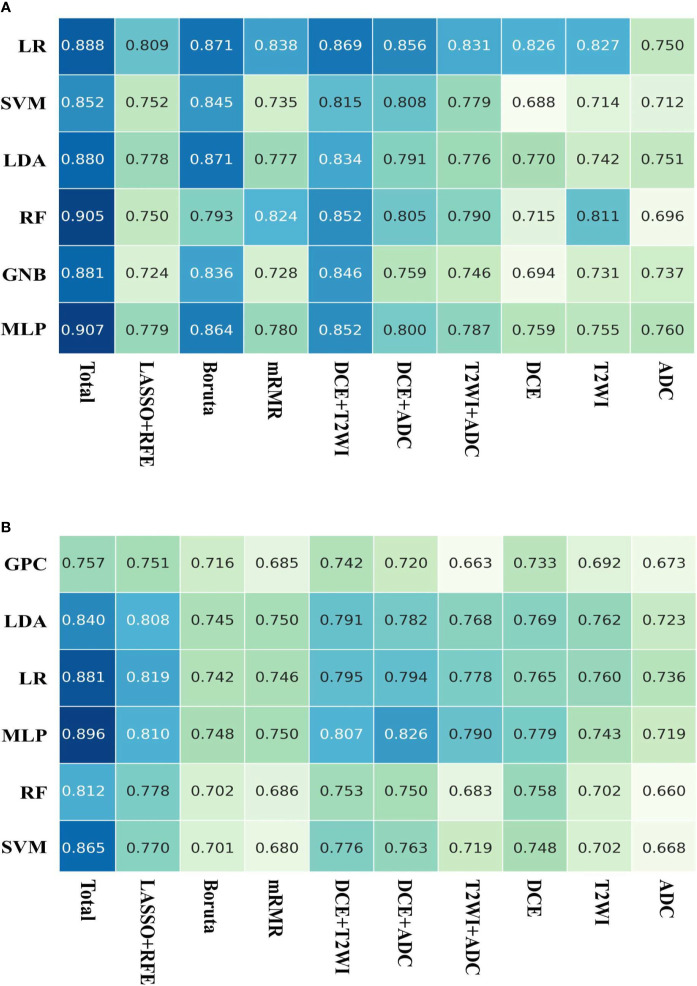
AUCs of the models on the testing dataset. **(A)** Six machine learning classifiers and 10 feature sets were utilized for predictive model construction on predicting AR expression; **(B)** six machine learning classifiers and 10 feature sets were utilized for predictive model construction on predicting molecular subtype.

Based on the LOOCV feature selection strategy, we constructed different machine models with distinct feature sets and algorithms. The AUCs of these models are shown in **Figure 4**. For predicting molecular subtype, the mean AUC of models using the total features was 0.842, which outperformed that of the models with other nine feature sets. The feature sets from three selection strategies had various mean AUCs, and the LASSO-RFE was better than other two selection strategies (mean micro-AUC: 0.789, 0.726, and 0.716 in LASSO-RFE, Boruta, and mRMR, respectively). While to assess the role of feature sets from different MRI in model performance, we compared different combinations of features from DCE-MRI, T2WI, and ADC-map. When using the total features, the models had the best performance. For predicting AR expression, the mean AUC of models using the total features was 0.886, which outperformed that of the models with other nine feature sets as well. The Boruta-based feature set outperformed other two feature sets (mean micro-AUC: 0.847, 0.780, and 0.765 in Boruta, mRMR, and LASSO-RFE, respectively). And the models using the total feature had the better performance than other combinations of MRI sequences. So in the further machine learning model constructions, we used the total features so as to ensure a high performance of the models for predicting both molecular subtype and AR expression.

**Figure 5:**
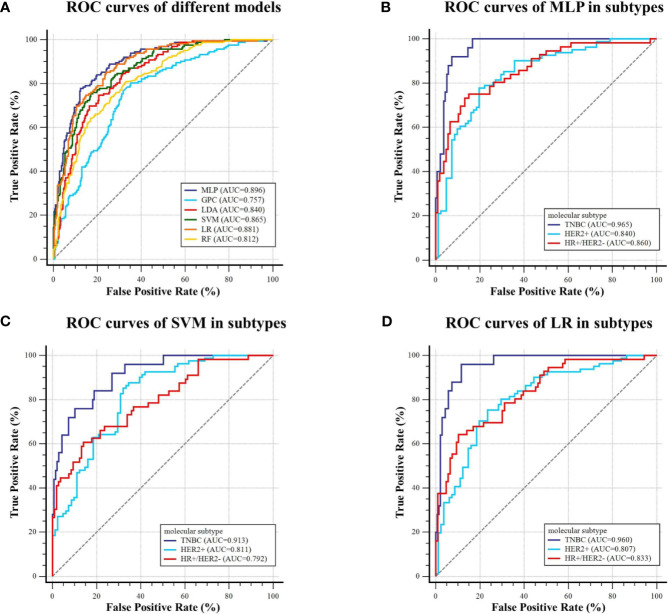
ROC curves of the models predicting molecular subtype on the testing dataset. **(A)** Six machine learning classifiers were utilized for predictive model construction and their AUCs; **(B–D)** ROC curve of MLP, SVM, and LR in classifying TNBC and non-TNBC, HER2+ and HER2−, and HR+/HER2− and others, respectively.

For predicting molecular subtype, the model performance for classifying TNBC *vs.* non-TNBC, HER2+ *vs.* HER2−, and HR+/HER2− *vs.* others in the testing dataset are shown in **Table 4**, and the micro-AUC and macro-AUC were also calculated to compare the model performances. To evaluate the performance of the multiclass model, precision, recall rate, F1-score, and overall accuracy were calculated in different subtypes, and the results are shown in **Table 5**. The micro-AUC values of MLP, GPC, LDA, SVM, RF, and LR model for predicting molecular subtype of breast cancer were 0.896, 0.757, 0.840, 0.865, 0.812, and 0.881, respectively. The MLP and LR model had the relatively highest AUCs, and these two AUCs were not significantly different (p-value = 0.119). The MLP also presented a considerable accuracy with 0.735, which was higher than other models. The other discriminative metrics also revealed a great diagnostic performance of the MLP model in the testing dataset. Then we evaluated the model performance in specific subtype, and the AUC (0.965; 95% CI: 0.924–0.987) of MLP model outperformed other models in classifying TNBC *vs.* non-TNBC, the accuracy was 92.6%, the sensitivity was 92.0%, and the specificity was 92.7%. The MLP model was also better than other models in classifying HER2+ *vs.* HER2−, and the AUC, accuracy, sensitivity, and specificity were 0.840 (95% CI: 0.774–0.893) and 79.0%, 77.8%, and 80.3%, respectively. For classifying HR+/HER2− *vs.* others, the MLP model had the highest AUC (0.860, 95% CI: 0.797–0.910), and its accuracy was 82.1%, with a sensitivity of 73.2% and a specificity of 86.8%. The ROC curves of various models are shown in **Figure 5**.

**Table 4:** Performances of the six machine learning classifiers for predicting molecular subtype.

Classifier	TNBC *vs.* non-TNBC		HR+/HER2− *vs.* Others		HER2+ *vs.* HER2−		micro-AUC	macro-AUC
	AUC	ACC (%)	AUC	ACC (%)	AUC	ACC (%)		
MLP	0.965	92.6	0.860	82.1	0.840	79.0	0.896	0.888
GPC	0.832	87.7	0.645	64.2	0.675	66.7	0.757	0.717
LDA	0.953	88.9	0.764	67.9	0.745	71.6	0.840	0.821
SVM	0.913	87.7	0.792	77.2	0.811	76.5	0.865	0.839
RF	0.897	78.4	0.726	75.3	0.714	67.9	0.812	0.779
LR	0.960	89.5	0.833	80.9	0.807	75.9	0.881	0.867

SVM, Support Vector Machine (radial bias function); RF, Random Forest; LR, Logistic Regression; LDA, Linear Discriminant Analysis; GPC, Gaussian Process Classifier; MLP, Multilayer Perceptron; AUC, the area under curve; ACC, accuracy; TNBC, triple-negative breast cancer; HR, hormone receptor; HER2, human epidermal growth factor receptor-2.

**Table 5:** Performances of the six machine learning classifiers for predicting molecular subtype.

Classifier	Subtype	Precision	Recall	F1-score	Overall Accuracy
MLP	TNBC	0.813	0.520	0.634	0.735
	HR+/HER2−	0.771	0.661	0.712	
	HER2+	0.704	0.852	0.771	
GPC	TNBC	0.692	0.360	0.474	0.623
	HR+/HER2−	0.628	0.482	0.545	
	HER2+	0.613	0.802	0.695	
LDA	TNBC	0.714	0.400	0.513	0.642
	HR+/HER2−	0.674	0.518	0.586	
	HER2+	0.619	0.802	0.699	
SVM	TNBC	0.778	0.280	0.412	0.623
	HR+/HER2−	0.697	0.411	0.517	
	HER2+	0.592	0.877	0.707	
RF	TNBC	0.625	0.400	0.488	0.636
	HR+/HER2−	0.628	0.482	0.545	
	HER2+	0.641	0.815	0.718	
LR	TNBC	0.733	0.440	0.550	0.679
	HR+/HER2−	0.700	0.625	0.660	
	HER2+	0.660	0.790	0.719	

SVM, Support Vector Machine (radial bias function); RF, Random Forest; LR, Logistic Regression; LDA, Linear Discriminant Analysis; GPC, Gaussian Process Classifier; MLP, Multilayer Perceptron; TNBC, triple-negative breast cancer; HR, hormone receptor; HER2, human epidermal growth factor receptor-2.

**Figure 6:**
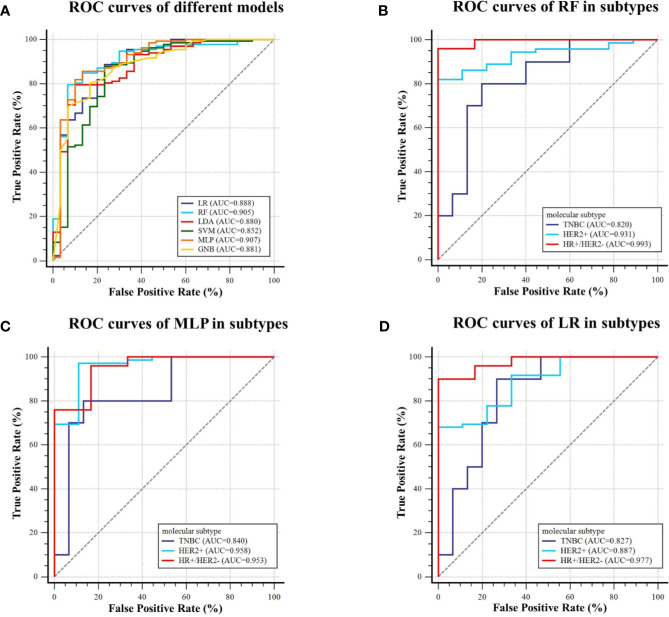
ROC curves of the models predicting AR expression on the testing dataset. **(A)** Six machine learning classifiers were utilized for predictive model construction and their AUCs; **(B–D)** ROC curve of RF, MLP, and LR in three subtypes (TNBC, HER2+, and HR+/HER2−), respectively.

**Figure 7:**
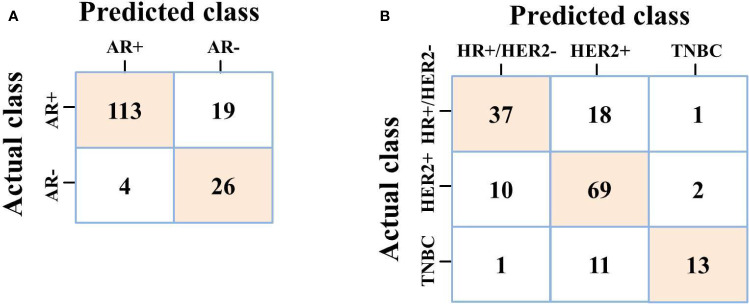
**(A)** Confusion matrix of MLP on predicting AR expression; **(B)** Confusion matrix of MLP on predicting molecular subtype.

For predicting AR expression, we compared the predictive performance of six models based on different classification algorithms in the testing dataset (see **Table 6**). The MLP model had an AUC of 0.907 and an accuracy of 85.8% in the testing dataset, which outperformed the other models. The model also showed a sensitivity of 85.6% and a specificity of 86.7% for predicting high AR expression. The ROC curves of different models in the testing dataset are shown in **Figure 6**. Using MLP algorithms to integrate the selected radiomics features, our models achieved the perfect AUCs to predict molecular subtype and AR expression, and the confusion matrixes are shown in **Figure 7**.

**Table 6:** Performances of the six machine learning classifiers for predicting AR expression.

Classifier	AUC (95% CI)	ACC (%)	SEN (%)	SPE (%)	NPV (%)	PPV (%)
MLP	0.907 (0.851–0.947)	85.8	85.6	86.7	57.8	96.6
LDA	0.880 (0.820–0.926)	81.5	79.6	90.0	50.0	97.2
SVM	0.852 (0.788–0.903)	85.8	87.9	76.7	59.0	94.3
GNB	0.881 (0.821–0.927)	80.9	80.3	83.3	49.0	95.5
RF	0.905 (0.849–0.945)	82.1	79.6	93.3	50.9	98.1
LR	0.888 (0.829–0.932)	86.4	88.6	76.7	60.5	94.4

SVM, Support Vector Machine (radial bias function); RF, Random Forest; LR, Logistic Regression; MLP, Multilayer Perceptron; GNB, Gaussian Naïve Bayes; LDA, Linear Discriminant Analysis; AUC, the area under curve; ACC, accuracy; SEN, sensitivity; SPE, specificity; NPV, negative predictive value; PPV, positive predictive value; AR, androgen receptor.

### Explanation of Features

Actually, it is still a challenge to compare the radiomics features extracted from macroscopic resolution in medical images to subcellular scale in histologic images, which could not provide direct biological explanation of radiomics features. However, the local comparisons and analysis could provide additional information of the radiomics features correlated to the observed histological signatures, thus enabling further screening of radiologic predictors for differentiating histological phenotype in a non-invasive way. We tried to explain the selected features on how they differentiated AR+ and AR− groups (and different molecular subtypes), and we used the inter-group distribution of features and PDP to reveal the marginal effects of radiomics features on the predicted labels. Several representative features from various MRI sequences were chosen to draw PDPs (see **Supplementary Material**). For predicting AR expression, a higher DCE_wavelet-HLH_firstorder_Kurtosis value was positively correlated with AR+, and such associations were consistent in distinct value ranges, and the distribution difference of such feature in AR+ and AR− also supported this finding (AR+: 6.343 ± 1.555; AR−: 5.114 ± 1.633). While a lower DCE_original_firstorder_Skewness value was inversely correlated with AR+ status, and the mean value of such feature in AR+ group was lower than that in AR− group actually (AR+: 0.088 ± 0.363; AR−: 0.254 ± 0.347). For features from T2WI and ADC-map, T2WI_wavelet-HLH_firstorder_Minimum, T2WI_wavelet-HLH_glcm_Contrast, and ADC_wavelet-HHH_ngtdm_Busyness also showed inverse correlation with AR+ status, while T2WI_wavelet-LLH_gldm _DependenceEntropy and ADC_original_firstorder_Kurtosis showed positive correlation with AR+ status. For predicting molecular subtypes, PDPs also provided a proper explanation. As shown in **Supplementary Material**, we chose three representative features from different MRI sequences and explored the relation between features and subtypes. The distributions of DCE_wavelet-LHH_glcm_Idn value in subtypes were as follows: 0.928 (HR+/HER2−) > 0.909 (HER2+) > 0.883 (TNBC), in which the PDPs also indicated a similar result. The distribution of T2WI_wavelet-HHH_glcm_Correlation and ADC_wavelet-HHL_glcm_ClusterShade values in three subtypes was also consistent with the PDP analysis [for T2WI_wavelet-HHH_glcm_Correlation: 0.059 (HER2+) > 0.053 (TNBC) > 0.045 (HR+/HER2−); for ADC_wavelet-HHL_glcm _ClusterShade: 0.569 (HR+/HER2−) > −0.021 (TNBC) > −0.551 (HER2+)]. Those findings revealed that some radiomics features were associated with AR expression and molecular subtypes. Taken together, these visualized PDPs could provide explanations of the selected features on how they influenced the predicted labels.

**Figure 8:**
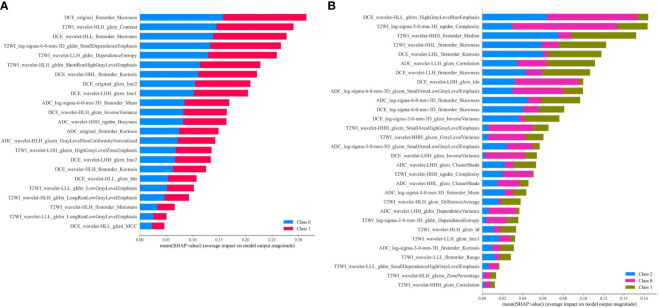
**(A)** Feature contribution weights for the MLP model predicting AR expression, and class 0 means AR < 10%, class 1 means AR ≥ 10%; **(B)** Feature contribution weights for the MLP model predicting molecular subtype, and class 0 means TNBC, class 1 means HER2+, class 2 means HR+/HER2−.

Furthermore, SHAP values were calculated of the two selected radiomics feature sets using the MLP models with the best AUC to visualize the importance rank of features, respectively. All the features with their importance degree contributing to prediction results are listed in **Figure 8**. From the feature ranking, we could know that different kinds of features had various influences on the predicted labels. For predicting AR expression, all the features played relatively equal roles in this binary classification model. While for predicting molecular subtype, some features had different effects on the predicted labels in MLP model.

## Discussion

Our study showed that some specific radiomics features extracted from multi-parametric MRI could predict molecular subtype and AR status in breast cancer. We further developed machine learning models with those selected radiomics features to differentiate molecular subtype and AR expression in breast cancer. For predicting molecular subtype, we used six classification algorithms to construct the model. The MLP classifiers showed the best diagnostic performance with the AUCs of 0.965, 0.840, and 0.860 and the accuracies of 92.6%, 79.0%, and 82.1% in three classification tasks (TNBC *vs.* non-TNBC, HER2+ *vs.* HER2−, and HR+/HER2− *vs.* others) on the testing dataset, respectively. And the micro-AUC (value: 0.896) and macro-AUC (value: 0.888) of the MLP model also outperformed other models, which indicated that MLP classifier had the great potential to assess molecular subtype. For predicting AR expression, the MLP classifier yielded the best performance with an AUC of 0.907 and an accuracy of 85.8% on the testing dataset. Some MRI-based radiomics predictors could non-invasively predict molecular subtype and AR expression in breast cancer. They may have tremendous potential to help with clinical diagnosis and treatment decisions.

The molecular subtypes of breast cancer could help to determine patients’ treatment selection and decision-making in clinical practice, such as endocrine therapy, anti-HER2 therapy, and cytotoxic therapy for different subtypes. TNBC is the most aggressive subtype of breast cancer and correlated with a worse prognosis than other subtypes. Patients with HR+ subtype cancer could receive endocrine therapy in addition to surgery and radiation treatment; patients with HER2+ subtype cancer could receive additional targeted treatment with monoclonal antibodies; patients with TNBC currently have no available targeted treatment (48). In recent years, AR has been proved to be widely expressed in breast cancer and was considered as a significant prognostic biomarker and therapeutic target in breast cancer (12, 16). The high AR expression was a significant independent prognostic factor that correlated to improved OS and DFS of breast cancer patients (12). Kevin H Kensler et al. have reported that AR+ indicated a better prognosis in ER+ tumors and a poor prognosis in ER− tumors (14). For TNBC, a recent study revealed that AR+/Cath-D+ co-expression was an independent prognostic factor of worse OS, and AR and Cath-D may be the therapeutic targets for combinatory therapy (49). For breast cancer patients treated with neoadjuvant chemotherapy, Isabell Witzel et al. found that a high AR mRNA level was associated with a lower rate of pathological complete remission (pCR) but with an improved prognosis, including DFS and OS in the neoadjuvant TECHNO clinical trials (17). The above studies indicated that AR could be regarded as a candidate biomarker for breast cancer. In our study, significant distribution differences of ER, PR, and HER2 expression were found between the low-AR group and the high-AR group, and the p-values were <0.001, <0.001, and 0.015, respectively, which contrasted to previous research and indicated interaction of protein expression might exist among those receptors in breast cancer. Due to the therapeutic and prognostic value, more attentions have been given to the study of AR expression in breast cancer in clinical practice.

Jiande Wu et al. reported that machine learning analysis of RNA-Sequence data was efficient and could be used for classifying breast cancer into TNBC and non-TNBC (39). Compared with gene detection, the histologic results from immunohistochemistry were easily obtained with lower spending, and it was used widely in clinical practice. However, obtaining breast cancer tissue was an invasive operation and might be inapplicable for some patients. Due to the tumoral volume limitation, location difficulty of puncture, non-standard IHC, or other inapplicable situations for biopsy histology, a traditional breast biopsy may not accurately reflect the molecular subtype and the degree of AR expression. Our study hypothesized that the spatial heterogeneity of breast tumor differed among molecular subtypes and could be quantified using radiomic method. To derive sufficient information from the entire tumor in a non-invasive way, our study used different machine learning algorithms combining distinct radiomics feature sets to find the optimal models to differentiate TNBC *vs.* non-TNBC, HER2+ *vs.* HER2−, and HR+/HER2− *vs.* others, and achieved excellent performance. We also found that radiomics features derived from three MRI sequences were all critical for model construction since the combination of feature sets from multi-parametric MRI had the best performance in the prediction model. Therefore, the radiomics-based models that we constructed had the potential to make a differential diagnosis of molecular subtype of breast cancer. We also explored a non-invasive radiomics-based method to detect AR expression, which might help develop a more comprehensive treatment strategy for patients with breast cancer.

Breast multi-parametric MRI (including T1-DCE, DWI, et al.) is increasingly applied in clinical routine imaging examination and is used for tumor diagnosis and response assessment (50). But it is difficult to adequately differentiate molecular subtypes of breast cancer by routine visual observing of medical images. Radiomics has been used as a quantitative analysis method for the correlation between image-based radiomics features and protein expression levels, which can provide comprehensive and objective information on tumoral biologic characteristics (26, 27). Several studies have shown that radiomics features from medical images have a great potential to be the surrogate marker for breast cancer phenotype (37, 38, 40, 43, 44, 51). Ming Fan et al. reported that a radiomics model based on the intra-tumoral and peri-tumoral heterogeneity in the decomposition of image time-series signals could accurately identify breast cancer subtypes with an AUC of 0.897 (43). Daniele La Forgia et al. reported that radiomics combining contrast-enhanced spectral mammography performed well in predicting histological subtypes of breast cancer, with accuracies of 90.87%, 83.79%, and 84.80% in discriminating HER2+/HER2−, ER+/ER−, and Ki67+/Ki67− breast cancer, respectively (44). Doris Leithner et al. found that a multi-parametric MRI-based radiomics approach might assist in the non-invasive differentiation of TN and luminal A subtype breast cancers from other subtypes (40). Those studies indicated that radiomics analysis was a useful analytical tool to predict receptor status in breast cancer. Previous studies have reported that some intra-tumoral radiomic biomarkers were associated with the histological characteristics of breast cancer (52–55). Actually, MRI-based radiomics required accurate tumor boundary labeling, which is a necessary step for traditional radiomics analysis. In addition, MRI-based radiomics could make better use of the spatial information of the whole tumor, which was ignored by breast ultrasound and mammography as they only reflected the tumor on a single image in most cases. The predictive outcomes of those studies included ER, PR, HER2 status, and Ki-67 expression, and radiomics models had been proven to have potential in detecting them. However, the feasibility of multi-parametric MRI-based radiomics analysis in predicting molecular subtype and AR status of breast cancer still needs to be verified.

There were some studies exploring the biological meaning of radiomics features, but no study had solved the problem (56–58). In our study, we tried to use PDPs to explain which relation the radiomics feature had with the histological outcomes. And we chose some representative features from multi-parametric MRI to draw PDPs. For predicting molecular subtype or AR expression, the PDPs of features from T1-DCE, T2WI, and ADC-map were all consistent with the distribution of the features in the groups. These findings suggested that some features extracted from medical images might relate to the phenotype of breast cancer. Though it is still hard to explain the biological foundations of these features, our study provided additional validation of radiomics features for relationships to histologic phenotypes.

In our study, breast cancers with various molecular subtypes were included for analysis. We used LOOCV to balance the covariates. After feature selection, 30 and 23 radiomics features were selected for predicting molecular subtype and AR expression, respectively. Those radiomics features were used to construct the predictive model with various classical classification algorithms. Of our models, MLP classifier had the best comprehensive performance in the differentiation of molecular subtype and AR expression. It indicated neural network model might have the great potential to be applied in the field of radiomics due to its strong processing ability in high-throughput data. Otherwise, we compared whether feature sets from three feature selection strategies and seven feature sets from different types and combinations of MRI sequences had various influences to the model construction. Those various feature sets actually developed models with different performance, and the combination of different types of features could improve the models. It also indicated that a multiple-feature selection strategy might be important for radiomics study. The results of our study indicated that our radiomics model might predict molecular subtype and AR expression non-invasively and significantly avoid unnecessary biopsy for breast cancer patients. Although we acknowledge that it is impossible to identify molecular expression using MR imaging alone accurately, underlying tumoral radiologic features may be associated with molecular expression, such as enhancement pattern and a lower ADC value (59). These tumoral imaging patterns could be easily visualized and quantified using radiomics approach (60). Our predictive model can differentiate molecular subtype and AR status in breast cancer non-invasively and guide individualized treatment. To the best of our knowledge, this is the first study to apply radiomics analysis to investigate the correlation between MRI image-derived radiomics features and AR expression in breast cancer. And we explored the application value of multi-parametric MRI-based radiomics model in predicting molecular subtypes of breast cancer.

There are some limitations of our study. First, the sample size for analysis was small, and the proportion of low-AR expression patients and TNBC were relatively small. Although we corrected the performance of model by LOOCV and eventually achieved a promising result, such imbalance might influence the machine learning model development. Second, our study was retrospective and needed to be validated with an external cohort to determine the value of our model in clinical practice and improve the confidence of performance. Third, only MR image radiomics features were used for analysis. Multi-omics study combining other medical images, such as ultrasound images, mammography, or breast CT, might improve the performance of our predictive model. Compared to other published studies, an integrated model using different types of medical images is worth further study, and our study only analyzed MRI-derived radiomics features. Fourth, we just used classical supervised classification algorithm to construct models, and deep learning–based features from MR images need further study. Our next step is to conduct prospective and standardized research on the multi-omics study for predicting AR expression and molecular subtype. And external validations from multi-centers will be considered in our future study.

## Conclusions

Our study explored the feasibility of the MRI-based radiomics features for predicting the molecular subtype and AR expression of breast cancer. Some radiomics features were associated with the expression of receptors in breast cancer and might have a predictive value. A radiomics model based on the selected radiomics features was constructed to assess the molecular subtype and histological AR status for individual breast cancer patients non-invasively and achieved a great performance. Our model could serve as an efficient tool to assist in clinical decision-making process.

## Data Availability Statement

The raw data supporting the conclusions of this article will be made available by the authors, without undue reservation.

## Ethics Statement

The studies involving human participants were reviewed and approved by the Ethical Committee of the First Affiliated Hospital, Sun Yat-sen University. Written informed consent for participation was not required for this study in accordance with the national legislation and the institutional requirements. Written informed consent was obtained from the individual(s) for the publication of any potentially identifiable images or data included in this article.

## Author Contributions

YHH contributed to concept development, study management, data curation, literature searching, and writing original draft. LW contributed to the analysis of pathology and writing original draft. YLH contributed to study management, literature searching, methodology, software using, and language editing. SH contributed to the data curation, imaging processing, and methodology. NS contributed to the concept development, study management, and writing original draft. YYL contributed to the MR imaging processing. HS contributed to the analysis of pathology. XZ contributed to the analysis of breast imaging and writing original draft. YL contributed to the concept development, funding acquisition, and writing original draft. All authors contributed to the critical review. All authors contributed to the article and approved the submitted version.

## Funding

This project was funded by the Sun Yat-Sen University Clinical Research 5010 Program (2016007). The funder played no role in determining the content of the manuscript or in the decision regarding whether to submit the manuscript for publication.

## Conflict of Interest

The authors declare that the research was conducted in the absence of any commercial or financial relationships that could be construed as a potential conflict of interest.

The reviewer ZC declared a shared affiliation with the authors to the handling editor at time of review.

## Publisher’s Note

All claims expressed in this article are solely those of the authors and do not necessarily represent those of their affiliated organizations, or those of the publisher, the editors and the reviewers. Any product that may be evaluated in this article, or claim that may be made by its manufacturer, is not guaranteed or endorsed by the publisher.

## Abbreviations

ER, estrogen receptor; PR, progesterone receptor; HER2, human epidermal growth factor receptor-2; TNBC, triple-negative breast cancer; HR, hormonal receptor; MRI, magnetic resonance imaging; T1-DCE, T1-weighted dynamic contrast-enhanced images; DWI, diffusion-weighted images; FS-T2WI, fat-suppressed T2-weighting images; ADC, apparent dispersion coefficient; IHC, immunohistochemistry; FISH, fluorescence *in situ* hybridization; ROI, regions of interest; LOOCV, leave-one-out cross-validation; LASSO, the least absolute shrinkage and selection operator; RFE, recursive feature elimination; ROC, the receiver operation characteristic curves; AUC, the area under the receiver operating characteristic curve; pCR, pathological complete response; PCCM, Pearson correlation coefficient matrix; SD, standard deviation; 95% CI, 95% confidence interval; OS, overall survival; DFS, disease-free survival; LR, Logistic Regression; RF, Random Forest; SVM, Support Vector Machine; LDA, Linear Discriminant Analysis; GPC, Gaussian process classifier; GNB, Gaussian Naïve Bayes; MLP, Multilayer Perceptron; GLCM, gray level co-occurrence matrix; GLSZM, gray level size zone matrix; GLRLM, gray level run length matrix; NGTDM, neighborhood gray-tone difference matrix; NGLDM, neighboring gray level dependence matrix.
