# Supplementary material for: Multi-Parametric MRI-Based Radiomics Models for Predicting Molecular Subtype and Androgen Receptor Expression in Breast Cancer
Source: Front Oncol. 2021 Aug 18;11:706733. doi: 10.3389/fonc.2021.706733 (PMC8416497; doi:10.3389/fonc.2021.706733)
Supplement: Supplementary file 1 [file DataSheet_1.docx]

**Supplementary Material**

**Part 1: Radiomics Features Calculation**

1. **Shape-based features**

In this group of features, we included descriptors of the three-dimensional shape and size of the tumor region. Let in the following definitions V denote the volume and A the surface area of the volume of interest. We determined the following shape and size based features:

1. **Compactness 1**=$\frac{V}{\sqrt{\pi}A^{\frac{2}{3}}}$
2. **Compactness 2**=$36\pi\frac{V^{2}}{A^{3}}$
3. **Maximum 3d diameter**: The maximum three-dimensional tumor diameter is measured as the largest pairwise Euclidean distance, between voxels on the surface of the tumor volume.
4. **Spherical disproportion** = $\frac{A}{4\pi R^{2}}$
5. **Sphericity** =$\frac{\pi^{\frac{1}{3}}(6V)^{\frac{2}{3}}}{A}$
6. **Surface area**: The surface area is calculated by triangulation (i.e. dividing the surface into connected triangles) and is defined as:

$$A\text{=}\sum_{\text{i}=1}^{N} \frac{1}{2}\left|a_{i}b_{i}\times a_{i}c_{i}\right|$$

Where N is the total number of triangles covering the surface and a, b and c are edge vectors of the triangles.

1. **Surface to volume ratio** =$\frac{A}{V}$
2. **Volume**: The volume (V) of the tumor is determined by counting the number of pixels in the tumor region and multiplying this value by the voxel size.

**(2) First order statistical features**

The following 17 statistical features were extracted.

Let **X** be the three dimensional image matrix with *N* voxels of the ROI and P be the first order histogram distribution with *N_g_* discrete intensity levels.

1. **IntensityMax:** The maximum intensity value of **X.**
2. **IntensityMin:** The minimum intensity value of **X**.
3. **Median:** The median intensity value of **X**.
4. **IntensityStd:**

$$IntensityStd=(\frac{1}{L*W*H-1}\sum_{i=1}^{L} \sum_{j=1}^{W} \sum_{k=1}^{H} (X(\mathrm{i},\mathrm{j},\mathrm{k})-IntensityAve)^{2})^{1/2}$$

1. **Mean:**

$$\frac{1}{N}\sum_{i}^{N} X(i)$$

1. **Variance:**

$$\sqrt{\sum_{j=1}^{N_{g}} P(\mathrm{j})*(\mathrm{j}-\sum_{i=1}^{N_{g}} P(\mathrm{i})*\mathrm{i})^{2}}$$

1. **Skewness:**

$$\sqrt{\sum_{j=1}^{N_{g}} P(\mathrm{j})*(\mathrm{j}-\sum_{i=1}^{N_{g}} P(\mathrm{i})*\mathrm{i})^{3}}$$

1. **Kurtosis:**

$$\sqrt{\sum_{j=1}^{N_{g}} P(\mathrm{j})*(\mathrm{j}-\sum_{i=1}^{N_{g}} P(\mathrm{i})*\mathrm{i})^{4}}$$

1. **Range:**

The range of intensity values of **X**.

1. **Mean absolute deviation:**

The mean of the absolute deviations of all voxel intensities around the mean intensity value

1. **Energy:**

$$\sum_{i}^{N} X(i)^{2}$$

1. **Entropy:**

$$\sum_{i=1}^{N_{g}} P(i)\log_{2}P(i)$$

1. **Entropy_p:**

$$\sum_{i=1}^{N_{g}} \frac{P(i)}{N}\log_{2}\frac{P(i)}{N}$$

1. **Root mean square:**

$$\sqrt{\frac{\sum_{i}^{N}X(i)^{2}}{N}}$$

1. **Uniformity:**

$$\sum_{i=1}^{N_{g}} P(i)^{2}$$

1. **Uniformity_p:**

$$\sum_{i=1}^{N_{g}} (\frac{P(i)}{N})^{2}$$

1. **Mass:**

The sum intensity value of $X$.

**(3) Textural features**

Second order statistic texture features, and higher order statistic texture features were extracted. Forty-four second order statistic texture features could be calculated from the Gray Level Co-occurrence Matrix (GLCM). Forty-six high order statistic texture features were calculated from the Gray Level Size Zone Matrix (GLSZM), Gray Level Run Length Matrix (GLRLM), and Neighborhood Gray Tone Difference Matrix (NGTDM). All of the GLCM, GLSZM, GLRLM, and NGTDM based texture feature were calculated using a 2D analysis and then averaged for all slices within the three-dimensional tumor volume.

*Gray-Level Co-Occurrence Matrix based features (GLCM)*

GLCM based features were second-order statistical texture features, which are defined as a matrix *M* (*i, j; δ, θ*) to indicate the relative frequency with intensity values of pixels (*i* and *j*) at the distance of *δ* in direction *θ*.

Let:

*M*(*i, j*) be the co-occurrence matrix for an arbitrary *δ* and *θ*, set *δ=1 and θ=0 and 45*

*N_g_* be the number of discrete intensity levels in the images, set as 25,

*μ* be the mean of *M*(*i, j*),

$m_{x}(\mathrm{i})=\sum_{j=1}^{N_{g}} M(\mathrm{i},\mathrm{j})$ be the marginal row probabilities,

$m_{y}(\mathrm{i})=\sum_{i=1}^{N_{g}} M(\mathrm{i},\mathrm{j})$ be the marginal column probabilities, and *u_y ,_μ_x,_* be the mean of *m_x_* .and *m_y_*

$HX=-\sum_{i=1}^{N_{g}} m_{x}(i)log(m_{x}(i)$,

$HY=-\sum_{i=1}^{N_{g}} m_{y}(i)log(m_{y}(i)$,

$HXY=-\sum_{i=1}^{N_{g}} \sum_{j=1}^{N_{g}} m(i,j)log(m(i,j))$,

$HXY1=-\sum_{i=1}^{N_{g}} \sum_{j=1}^{N_{g}} m(i,j)log(m_{x}(i)m_{y}(j))$.

$HXY2=-\sum_{i=1}^{N_{g}} \sum_{j=1}^{N_{g}} m_{x}(i)m_{y}(j)log(m_{x}(i)m_{y}(j))$.

1. **Energy:**

$$\sum_{i=1}^{N_{g}} \sum_{j=1}^{N_{g}} [\mathrm{M}(\mathrm{i},\mathrm{j})]^{2}$$

1. **Contrast:**

$$\sum_{i=1}^{N_{g}} \sum_{j=1}^{N_{g}} (\mathrm{i}-\mathrm{j})^{2}*M(\mathrm{i},\mathrm{j})$$

1. **Entropy:**

$$\sum_{i=1}^{N_{g}} \sum_{j=1}^{N_{g}} M(\mathrm{i},\mathrm{j})*\log_{2}M(\mathrm{i},\mathrm{j})$$

1. **Homogeneity 1:**

$$\sum_{i=1}^{N_{g}} \sum_{j=1}^{N_{g}} \frac{M(\mathrm{i},\mathrm{j})}{1+|\mathrm{i}-\mathrm{j}|}$$

1. **Homogeneity 2:**

$$\sum_{i=1}^{N_{g}} \sum_{j=1}^{N_{g}} \frac{M(\mathrm{i},\mathrm{j})}{1+|\mathrm{i}-\mathrm{j}|^{2}}$$

1. **Correlation:**

$$\frac{\sum_{i=1}^{N_{g}} \sum_{j=1}^{N_{g}} ijM(\mathrm{i},\mathrm{j})-\mu_{i}(\mathrm{i})\mu_{j}(\mathrm{j})}{\sigma_{x}(\mathrm{i})\sigma_{y}(\mathrm{j})}$$

1. **Variance:**

$$\sum_{i=1}^{N_{g}} \sum_{j=1}^{N_{g}} (\mathrm{i}-\mu)^{2}M(\mathrm{i},\mathrm{j})$$

1. **Sum Average:**

$$\sum_{i=2}^{2N_{g}} iM_{x+y}(\mathrm{i})$$

1. **Sum Entropy:**

$$-\sum_{i=2}^{2N_{g}} M_{x+y}(\mathrm{i})\log_{2}\left[M_{x+y}(\mathrm{i})\right]$$

1. **Dissimilarity:**

$$\sum_{i=1}^{N_{g}} \sum_{j=1}^{N_{g}} |\mathrm{i}-\mathrm{j}|\mathrm{M}(\mathrm{i},\mathrm{j})$$

1. **Inverse Difference Moment:**

$$\sum_{i=1}^{N_{g}} \sum_{j=1}^{N_{g}} \frac{\mathrm{M}(\mathrm{i},\mathrm{j})}{1+(\frac{|\mathrm{i}-\mathrm{j}|^{2}}{N^{2}})}$$

1. **Autocorrelation:**

$$\sum_{i=1}^{N_{g}} \sum_{j=1}^{N_{g}} ijM(\mathrm{i},\mathrm{j})$$

1. **Cluster Prominence**

$$\sum_{i=1}^{N_{g}} \sum_{j=1}^{N_{g}} \left[i+j-u_{x}-u_{y}\right]^{4}M(\mathrm{i},\mathrm{j})$$

1. **Cluster Shade**

$$\sum_{i=1}^{N_{g}} \sum_{j=1}^{N_{g}} \left[i+j-u_{x}-u_{y}\right]^{3}M(\mathrm{i},\mathrm{j})$$

1. **Cluster Tendency**

$$\sum_{i=1}^{N_{g}} \sum_{j=1}^{N_{g}} \left[i+j-u_{x}-u_{y}\right]^{2}M(\mathrm{i},\mathrm{j})$$

1. **Difference Entropy**

$$-\sum_{i=0}^{N_{g}-1} M_{x-y}(i)\log_{2}\left[M_{x-y}(i)\right]$$

1. **Maximum Probability:**

$$\max\left\{M(i,j)\right\}$$

1. **Sum variance**

$$\sum_{i=2}^{2N_{g}} (i-SE)^{2}M_{x+y}(i)$$

1. **Informational measure of correlation 1 (IMC1):**

$$\frac{HXY-HXY1}{\max\left\{HX-HY\right\}}$$

1. **Informational measure of correlation 2 (IMC2):**

$$\sqrt{1-\text{e}^{-2(HXY2-HXY)}}$$

1. **Inverse Difference Moment Normalized (IDMN):**

$$\sum_{i=1}^{N_{g}} \sum_{j=1}^{N_{g}} \frac{M(i,j)}{1+(\frac{\left|i-j\right|^{2}}{N^{2}})}$$

1. **Inverse Difference Normalized (IDN):**

$$\sum_{i=1}^{N_{g}} \sum_{j=1}^{N_{g}} \frac{M(i,j)}{1+(\frac{\left|i-j\right|}{N^{2}})}$$

*Gray Level Run Length Matrix based features (GLRLM)*

GLRLM based features were high-order statistical texture feature, which were defined as *P*(*i, j; θ*) to indicate the number of times j and gray level i appear consecutively in the direction *θ*.

Let:

*P*(*i, j; θ*) be the run-length matrix *P* for a direction *θ*, set *θ=0 and 45*

*N_g_* be the number of discrete intensity values,

*N_r_* be the number of different run lengths, and

*N_p_* be the number of voxels in the ROI.

1. **Short Run Emphasis (SRE):**

$$\frac{\sum_{i=1}^{N_{g}} \sum_{j=1}^{N_{r}} [\frac{P(\mathrm{i},\mathrm{j};\theta)}{j^{2}}]}{\sum_{i=1}^{N_{g}} \sum_{j=1}^{N_{r}} P(\mathrm{i},\mathrm{j};\theta)}$$

1. **Long Run Emphasis (LRE):**

$$\frac{\sum_{i=1}^{N_{g}} \sum_{j=1}^{N_{r}} j^{2}P(\mathrm{i},\mathrm{j};\theta)}{\sum_{i=1}^{N_{g}} \sum_{j=1}^{N_{r}} P(\mathrm{i},\mathrm{j};\theta)}$$

1. **Gray-Level Nonuniformity (GLN):**

$$\frac{\sum_{i=1}^{N_{g}} [\sum_{j=1}^{N_{r}} P(\mathrm{i},\mathrm{j};\theta)]^{2}}{\sum_{i=1}^{N_{g}} \sum_{j=1}^{N_{r}} P(\mathrm{i},\mathrm{j};\theta)}$$

1. **Run-Length Nonuniformity (RLN):**

$$\frac{\sum_{j=1}^{N_{r}} [\sum_{i=1}^{N_{g}} P(\mathrm{i},\mathrm{j};\theta)]^{2}}{\sum_{i=1}^{N_{g}} \sum_{j=1}^{N_{r}} P(\mathrm{i},\mathrm{j};\theta)}$$

1. **Run Percentage (RP):**

$$\sum_{i=1}^{N_{g}} \sum_{j=1}^{N_{r}} \frac{P(\mathrm{i},\mathrm{j};\theta)}{N_{p}}$$

1. **Low Gray-Level Run Emphasis (LGRE):**

$$\frac{\sum_{i=1}^{N_{g}} \sum_{j=1}^{N_{r}} [\frac{P(\mathrm{i},\mathrm{j};\theta)}{i^{2}}]}{\sum_{i=1}^{N_{g}} \sum_{j=1}^{N_{r}} P(\mathrm{i},\mathrm{j};\theta)}$$

1. **High Gray-Level Run Emphasis (HGRE):**

$$\frac{\sum_{i=1}^{N_{g}} \sum_{j=1}^{N_{r}} i^{2}P(\mathrm{i},\mathrm{j};\theta)}{\sum_{i=1}^{N_{g}} \sum_{j=1}^{N_{r}} P(\mathrm{i},\mathrm{j};\theta)}$$

1. **Short Run Low Gray-Level Emphasis (SRLGE):**

$$\frac{\sum_{i=1}^{N_{g}} \sum_{j=1}^{N_{r}} \frac{P(\mathrm{i},\mathrm{j};\theta)}{i^{2}j^{2}}}{\sum_{i=1}^{N_{g}} \sum_{j=1}^{N_{r}} P(\mathrm{i},\mathrm{j};\theta)}$$

1. **Short Run High Gray-Level Emphasis (SRHGE):**

$$\frac{\sum_{i=1}^{N_{g}} \sum_{j=1}^{N_{r}} \frac{i^{2}P(\mathrm{i},\mathrm{j};\theta)}{j^{2}}}{\sum_{i=1}^{N_{g}} \sum_{j=1}^{N_{r}} P(\mathrm{i},\mathrm{j};\theta)}$$

1. **Long Run Low Gray-Level Emphasis (LRLGE):**

$$\frac{\sum_{i=1}^{N_{g}} \sum_{j=1}^{N_{r}} \frac{j^{2}P(\mathrm{i},\mathrm{j};\theta)}{i^{2}}}{\sum_{i=1}^{N_{g}} \sum_{j=1}^{N_{r}} P(\mathrm{i},\mathrm{j};\theta)}$$

1. **Long Run High Gray-Level Emphasis (LRHGE):**

$$\frac{\sum_{i=1}^{N_{g}} \sum_{j=1}^{N_{r}} i^{2}j^{2}P(\mathrm{i},\mathrm{j};\theta)}{\sum_{i=1}^{N_{g}} \sum_{j=1}^{N_{r}} P(\mathrm{i},\mathrm{j};\theta)}$$

1. **Mean:**

$$\frac{1}{2N_{\text{g}}}\sum_{i=1}^{N_{g}} \sum_{j=1}^{N_{g}} [P(\mathrm{i},\mathrm{j})]^{2}$$

1. **Entropy:**

$$\sum_{i=1}^{N_{g}} \sum_{j=1}^{N_{g}} P(\mathrm{i},\mathrm{j})*\log_{2}P(\mathrm{i},\mathrm{j})$$

1. **Energy:**

$$\sum_{i=1}^{N_{g}} \sum_{j=1}^{N_{g}} [P(\mathrm{i},\mathrm{j})]^{2}$$

*Gray Level Size Zone Matrix based features (GLSZM)*

GLSZM based features were high-order statistical texture features, which were defined as *P*(*i, j*) to indicate the areas of size j and gray level i.

Let:

*P*(*i, j*) be the size zone of matrix *P*,

*N_g_* be the number of discrete intensity values,

*N_r_* be the number of different areas sizes,

*N_p_* be the number of voxels in the ROI.

1. **Small Zone Emphasis (SZE):**

$$\frac{\sum_{i=1}^{N_{g}} \sum_{j=1}^{N_{r}} [\frac{P(\mathrm{i},\mathrm{j})}{j^{2}}]}{\sum_{i=1}^{N_{g}} \sum_{j=1}^{N_{r}} P(\mathrm{i},\mathrm{j})}$$

1. **Large Zone Emphasis (LZE):**

$$\frac{\sum_{i=1}^{N_{g}} \sum_{j=1}^{N_{r}} j^{2}P(\mathrm{i},\mathrm{j})}{\sum_{i=1}^{N_{g}} \sum_{j=1}^{N_{r}} P(\mathrm{i},\mathrm{j})}$$

1. **Gray-Level Nonuniformity (GLN):**

$$\frac{\sum_{i=1}^{N_{g}} [\sum_{j=1}^{N_{r}} P(\mathrm{i},\mathrm{j})]^{2}}{\sum_{i=1}^{N_{g}} \sum_{j=1}^{N_{r}} P(\mathrm{i},\mathrm{j})}$$

1. **Zone-Size Nonuniformity (ZSN):**

$$\frac{\sum_{j=1}^{N_{r}} [\sum_{i=1}^{N_{g}} P(\mathrm{i},\mathrm{j})]^{2}}{\sum_{i=1}^{N_{g}} \sum_{j=1}^{N_{r}} P(\mathrm{i},\mathrm{j})}$$

1. **Zone Percentage (ZP):**

$$\sum_{i=1}^{N_{g}} \sum_{j=1}^{N_{r}} \frac{P(\mathrm{i},\mathrm{j})}{N_{p}}$$

1. **Low Gray-Level Zone Emphasis (LGZE):**

$$\frac{\sum_{i=1}^{N_{g}} \sum_{j=1}^{N_{r}} [\frac{P(\mathrm{i},\mathrm{j})}{i^{2}}]}{\sum_{i=1}^{N_{g}} \sum_{j=1}^{N_{r}} P(\mathrm{i},\mathrm{j})}$$

1. **High Gray-Level Zone Emphasis (HGZE):**

$$\frac{\sum_{i=1}^{N_{g}} \sum_{j=1}^{N_{r}} i^{2}P(\mathrm{i},\mathrm{j})}{\sum_{i=1}^{N_{g}} \sum_{j=1}^{N_{r}} P(\mathrm{i},\mathrm{j})}$$

1. **Small Zone Low Gray-Level Emphasis (SZLGE):**

$$\frac{\sum_{i=1}^{N_{g}} \sum_{j=1}^{N_{r}} \frac{P(\mathrm{i},\mathrm{j})}{i^{2}j^{2}}}{\sum_{i=1}^{N_{g}} \sum_{j=1}^{N_{r}} P(\mathrm{i},\mathrm{j})}$$

1. **Small Zone High Gray-Level Emphasis (SZHGE):**

$$\frac{\sum_{i=1}^{N_{g}} \sum_{j=1}^{N_{r}} \frac{i^{2}P(\mathrm{i},\mathrm{j})}{j^{2}}}{\sum_{i=1}^{N_{g}} \sum_{j=1}^{N_{r}} P(\mathrm{i},\mathrm{j})}$$

1. **Large Zone Low Gray-Level Emphasis (LZLGE):**

$$\frac{\sum_{i=1}^{N_{g}} \sum_{j=1}^{N_{r}} \frac{j^{2}P(\mathrm{i},\mathrm{j})}{i^{2}}}{\sum_{i=1}^{N_{g}} \sum_{j=1}^{N_{r}} P(\mathrm{i},\mathrm{j})}$$

1. **Large Zone High Gray-Level Emphasis (LZHGE):**

$$\frac{\sum_{i=1}^{N_{g}} \sum_{j=1}^{N_{r}} i^{2}j^{2}P(\mathrm{i},\mathrm{j})}{\sum_{i=1}^{N_{g}} \sum_{j=1}^{N_{r}} P(\mathrm{i},\mathrm{j})}$$

1. **Gray-Level Variance (GLV):**

$$\sum_{i=1}^{N_{g}} \sum_{j=1}^{N_{r}} \{\mathrm{iP}(\mathrm{i},\mathrm{j})-\frac{\sum_{i=1}^{N_{g}} i[\sum_{j=1}^{N_{r}} P(\mathrm{i},\mathrm{j})]}{N_{g}N_{r}}\}$$

1. **Zone-Size Variance (ZSV):**

$$\sum_{i=1}^{N_{g}} \sum_{j=1}^{N_{r}} \{\mathrm{jP}(\mathrm{i},\mathrm{j})-\frac{\sum_{i=1}^{N_{g}} i[\sum_{j=1}^{N_{r}} P(\mathrm{i},\mathrm{j})]}{N_{g}N_{r}}\}$$

*Neighborhood Gray Tone Difference Matrix based features (NGTDM)*

NGTDM based features were high-order statistical texture features, which were defined as *S(i)* to indicate the sum of the absolute value between gray intensity level i and it’s neighbors’ average intensity.

Let:

*S(i)* be the sum of absolute value between gray intensity level i and its neighbors’ average intensity,

*C(i)* be the number of voxels with the gray intensity level I,

*N_g_* be the number of discrete intensity values.

1. **Coarseness:**

$$\frac{1}{\varepsilon+\sum_{i=1}^{N_{g}} \frac{C(\mathrm{i})\mathrm{S}(\mathrm{i})}{\sum_{i=1}^{N_{g}} C(\mathrm{i})}}$$

1. **Contrast:**

$$\frac{\sum_{i=1}^{N_{g}} \sum_{j=1}^{N_{g}} C(\mathrm{i})\mathrm{C}(\mathrm{j})(\mathrm{i}-\mathrm{j})^{2}}{(\sum_{i=1}^{N_{g}} C(\mathrm{i}))^{2}}*\sum_{i=1}^{N_{g}} S(\mathrm{i})*\frac{1}{N_{g}(\mathrm{N}_{g}-1)\sum_{i=1}^{N_{g}} C(\mathrm{i})}$$

1. **Busyness:**

$$\frac{\sum_{i=1}^{N_{g}} C(\mathrm{i})\mathrm{S}(\mathrm{i})}{\sum_{i=1}^{N_{g}} \sum_{j=1}^{N_{g}} (\mathrm{iC}(\mathrm{i})-\mathrm{jC}(\mathrm{j}))}, C(\mathrm{i})\neq0,\mathrm{C}(\mathrm{j})\neq0$$

1. **Complexity:**

$$\frac{1}{(\sum_{i=1}^{N_{g}} C(\mathrm{i}))^{2}}*\sum_{i=1}^{N_{g}} \sum_{j=1}^{N_{g}} \frac{|\mathrm{i}-\mathrm{j}|(\mathrm{C}(\mathrm{i})\mathrm{S}(\mathrm{i})+\mathrm{C}(\mathrm{j})\mathrm{S}(\mathrm{j}))}{(\mathrm{C}(\mathrm{i})+\mathrm{C}(\mathrm{j}))}, C(\mathrm{i})\neq0,\mathrm{C}(\mathrm{j})\neq0$$

1. **Strength:**

$$\frac{\sum_{i=1}^{N_{g}} \sum_{j=1}^{N_{g}} (\mathrm{C}(\mathrm{i})-\mathrm{C}(\mathrm{j}))*(\mathrm{i}-\mathrm{j})^{2}}{\sum_{i=1}^{N_{g}} C(\mathrm{i})*\sum_{i=1}^{N_{g}} S(\mathrm{i})}, C(\mathrm{i})\neq0,\mathrm{C}(\mathrm{j})\neq0$$

**(4) Wavelet features: first order statistical and texture features of a wavelet filtered image.**

A total of 1302 derived wavelet features were extracted for each sequence, with the Gaussian filter and a wavelet-based filter. These features were computed on the filtered images. The original image was filtered by 8 filters. For each image, the first order statistical and texture features were computed. Finally, 3906 wavelet-based features were extracted.

**Part 2: Feature Selection Algorithms**

**(1) LASSO (least absolute shrinkage and selection operator)**

The [Lasso](https://scikit-learn.org/stable/modules/generated/sklearn.linear_model.Lasso.html#sklearn.linear_model.Lasso) is a linear model that estimates sparse coefficients. It is useful in some contexts due to its tendency to prefer solutions with fewer non-zero coefficients, effectively reducing the number of features upon which the given solution is dependent. For this reason Lasso and its variants are fundamental to the field of compressed sensing. Under certain conditions, it can recover the exact set of non-zero coefficients. As the Lasso regression yields sparse models, it can thus be used to perform feature selection, as detailed in [L1-based feature selection](https://scikit-learn.org/stable/modules/feature_selection.html#l1-feature-selection).

**(2) RFE (recursive feature elimination)**

Given an external estimator that assigns weights to features (e.g., the coefficients of a linear model), the goal of recursive feature elimination (RFE) is to select features by recursively considering smaller and smaller sets of features. First, the estimator is trained on the initial set of features and the importance of each feature is obtained either through any specific attribute or callable. Then, the least important features are pruned from current set of features. That procedure is recursively repeated on the pruned set until the desired number of features to select is eventually reached.

**(3) mRMR (****maximum relevance minimum redundancy)**

The purpose of the mRMR is to select features that are most relevant to the predicted label. At the same time, the minimum redundancy process ensures minimum redundancy between the selected features to obtain the optimal features with a high correlation and a low redundancy. At our study, the retained number of feature was set as 30 at the initial feature selection step. At the subsequent analyses, the inter-group distribution of feature was analyzed, and for predicting AR expression, the AUC of each feature was also calculated to evaluate the importance of feature.

**(4) Boruta**

Boruta algorithm uses a wrapper method based on the RF classifier for feature selection. A “shadow” attribute was created for each feature in the feature pool by shuffling values of the original feature across all patients. Then the shadow attributes are combined with original features for classification using an RF model. The importance of shadow attribute is used as a reference for selecting truly important features, as determined by RF permutation importance measure. In the implementation of Boruta of our study, the number of trees for RF was set to 500 and the maximum number of importance source runs was set to 100.

**Part 3: Machine Learning Algorithms**

**(1) Random Forest**

Random forest consists of multiple decision trees. Random forest is a discriminant model that supports both classification and regression problems, and supports multiple classification problems. It is a nonlinear model. For classification problems, a test sample will be sent to each decision tree for prediction, and then voted, the category with the most votes is the final classification result. In addition, when performing node segmentation in the process of building a tree, the selected segmentation point is no longer the best segmentation point among all features, but the optimal segmentation point in a random subset of features. Due to this randomness, the deviation of the forest usually increases slightly (relative to the deviation of a single non-random tree), but because the average is taken, its variance will also decrease, and it can usually compensate for the increase in deviation, resulting in an overall better model. “n_estimators” and “criterion” were used in the tuning step of model development.

**(2) Logistics Regression**

Logistic regression is a linear regression model, which assumes that the data obey Bernoulli distribution. By means of maximum likelihood function, the gradient descent method is used to solve the parameters, so as to achieve the purpose of dichotomy.LR model can be considered as a linear regression model normalized by Sigmoid function (Logistic equation). Sigmoid compresses the data (LR, middle finger, output y) between [0, 1] and passes through an important point (0, 0.5). In this way, the output is compressed between [0,1], with 0.5 as the boundary value, 0.5 greater than 0.5 as one class, and 0.5 less than 0.5 as another class. “C”, “penalty” and “solver” were used in the tuning step of model development.

**(3) Naïve Bayes**

Naive Bayesian methods are a series of supervised learning methods based on the assumption that the application of Bayesian theory is naive and that each pair of characteristics is independent of each other. The main ideas of the Naive Bayes classifier: Through joint probability modeling, theorem of Bayes is used to solve the posterior probability; the category corresponding to the one with the largest posterior probability is used as the prediction category.

**(4) Support Vector Machine**

SVM mainly used to solve data classification problems in the field of pattern recognition, which belongs to a kind of supervised learning algorithm. It has strong nonlinear classification ability, showing many unique advantages in solving small sample, nonlinear and high-dimensional pattern recognition problems. “C” and “gamma” were used in the tuning step of model development.

**(5) Gaussian Process Classifier**

Gaussian process classification (GPC) based on Laplace approximation. Gaussian Processes (GP) are a generic supervised learning method designed to solve regression and probabilistic classification problems. The [Gaussian Process Classifier](https://scikit-learn.org/stable/modules/generated/sklearn.gaussian_process.GaussianProcessClassifier.html#sklearn.gaussian_process.GaussianProcessClassifier) implements Gaussian processes (GP) for classification purposes, more specifically for probabilistic classification, where test predictions take the form of class probabilities. Gaussian Process Classifier places a GP prior on a latent function f, which is then squashed through a link function to obtain the probabilistic classification.

**(6) Linear Discriminant Analysis**

Linear Discriminant Analysis and Quadratic Discriminant Analysis are two classic classifiers, with, as their names suggest, a linear and a quadratic decision surface, respectively. These classifiers are attractive because they have closed-form solutions that can be easily computed, are inherently multiclass, have proven to work well in practice, and have no hyper-parameters to tune. “solver” was used in the tuning step of model development.

**(7) Multilayer Perceptron**

MLP is a supervised learning algorithm that learns a function by training on a dataset. Given a set of features and a target, it can learn a non-linear function approximator for either classification or regression. It is different from logistic regression, in that between the input and the output layer, and there can be one or more non-linear layers, called hidden layers. MLP with hidden layers have a non-convex loss function where there exists more than one local minimum. Therefore different random weight initializations can lead to different validation accuracy. “hidden_layer_sizes”, “activation” and “solver” were used in the tuning step of model development.

**Supplementary Tables**

**Table S1.** The distribution and AUC value of retained radiomics features in classifying AR+ and AR- expression of breast cancer.

| **Feature name** | **Inter-group distribution (AR+ vs. AR-)** | | | **Performance** | |
| --- | --- | --- | --- | --- | --- |
|  | **AR+** | **AR-** | **p-value** | **AUC (95%CI)** | **p-value** |
| DCE_wavelet-HLL_glcm_Idn | 0.935±0.014 | 0.943±0.019 | 0.002 | 0.685 (0.607-0.755) | 0.005 |
| DCE_wavelet-HLL_glcm_MCC | 0.407±0.074 | 0.459±0.091 | 0.003 | 0.676 (0.598-0.747) | 0.004 |
| DCE_wavelet-LHH_glcm_Imc2 | 0.266±0.073 | 0.309±0.099 | 0.036 | 0.623 (0.544-0.698) | 0.052 |
| DCE_wavelet-LHH_glcm_Imc1 | -0.034±0.010 | -0.044±0.022 | 0.006 | 0.660 (0.582-0.733) | 0.006 |
| DCE_wavelet-HLH_firstorder_Kurtosis | 6.343±1.555 | 5.114±1.633 | 0.033 | 0.625 (0.546-0.700) | 0.048 |
| DCE_wavelet-HLL_firstorder_Skewness | -0.097±0.206 | -0.207±0.198 | 0.018 | 0.639 (0.560-0.713) | 0.008 |
| DCE_wavelet-HLH_glcm_InverseVariance | 0.466±0.032 | 0.442±0.055 | 0.011 | 0.649 (0.571-0.723) | 0.011 |
| DCE_original_glcm_Imc2 | 0.743±0.080 | 0.787±0.070 | 0.007 | 0.659 (0.580-0.731) | 0.003 |
| DCE_original_firstorder_Skewness | 0.088±0.363 | 0.254±0.347 | 0.024 | 0.630 (0.551-0.705) | 0.022 |
| DCE_wavelet-HHL_firstorder_Kurtosis | 4.716±1.057 | 5.356±1.699 | 0.111 | 0.593 (0.514-0.670) | 0.167 |
| T2WI_wavelet-HLH_glrlm_ShortRunHighGrayLevelEmphasis | 3.120±1.807 | 4.722±2.718 | <0.001 | 0.699 (0.622-0.768) | <0.001 |
| T2WI_wavelet-HLH_firstorder_Minimum | -42.803±17.172 | -30.370±22.473 | 0.003 | 0.677 (0.599-0.748) | 0.003 |
| T2WI_wavelet-LLL_glrlm_LongRunLowGrayLevelEmphasis | 0.026±0.065 | 0.012±0.024 | 0.012 | 0.647 (0.569-0.721) | 0.015 |
| T2WI_wavelet-HLH_glrlm_LongRunLowGrayLevelEmphasis | 2.231±1.468 | 1.521±1.288 | 0.002 | 0.684 (0.607-0.755) | 0.001 |
| T2WI_wavelet-LLH_gldm_DependenceEntropy | 6.018±0.323 | 5.896±0.354 | 0.011 | 0.648 (0.570-0.722) | 0.020 |
| T2WI_wavelet-LLL_gldm_LowGrayLevelEmphasis | 0.008±0.015 | 0.004±0.006 | 0.011 | 0.648 (0.569-0.722) | 0.015 |
| T2WI_wavelet-LHH_glszm_HighGrayLevelZoneEmphasis | 7.969±5.543 | 10.928±6.783 | 0.012 | 0.649 (0.570-0.722) | 0.016 |
| T2WI_wavelet-HLH_glcm_Contrast | 0.416±0.021 | 0.438±0.040 | <0.001 | 0.711 (0.635-0.780) | <0.001 |
| T2WI_log-sigma-6-0-mm-3D_gldm_SmallDependenceEmphasis | 0.007±0.003 | 0.009±0.007 | 0.010 | 0.650 (0.571-0.723) | 0.007 |
| ADC_log-sigma-6-0-mm-3D_firstorder_Mean | -118.98±91.28 | -77.09±48.28 | 0.003 | 0.676 (0.598-0.747) | <0.001 |
| ADC_wavelet-HHH_ngtdm_Busyness | 1812.2±2313.9 | 3218.9±4344.5 | 0.048 | 0.616 (0.537-0.691) | 0.047 |
| ADC_original_firstorder_Kurtosis | 3.435±0.864 | 3.083±0.926 | 0.006 | 0.662 (0.583-0.734) | 0.005 |
| ADC_wavelet-HLH_glszm_GrayLevelNonUniformityNormalized | 0.328±0.079 | 0.293±0.087 | 0.007 | 0.658 (0.579-0.731) | 0.012 |

Abbreviations: DCE, dynamic contrast enhancement; T2WI, T2-weighted imaging; ADC, apparent diffusion coefficient; AR, Androgen receptor; AUC, area under curve.

**Table S2.** The distribution of retained radiomics features in classifying molecular subtype of breast cancer.

| **Feature name** | **Inter-group distribution (TNBC vs. HER2+ vs. HR+/HER2-)** | | | |
| --- | --- | --- | --- | --- |
|  | **TNBC** | **HER2+** | **HR+/HER2-** | **p-value** |
| DCE_log-sigma-2-0-mm-3D_glcm_InverseVariance | 0.393±0.090 | 0.405±0.086 | 0.432±0.073 | 0.016 |
| DCE_wavelet-LLH_firstorder_Skewness | -0.164±0.128 | -0.031±0.186 | -0.113±0.228 | 0.110 |
| DCE_wavelet-HLL_glrlm_HighGrayLevelRunEmphasis | 606.83±582.78 | 818.85±352.67 | 284.81±300.46 | 0.123 |
| DCE_wavelet-LHL_firstorder_Kurtosis | 5.035±1.064 | 3.580±1.110 | 4.677±0.817 | 0.005 |
| DCE_wavelet-LHH_glcm_Idn | 0.883±0.037 | 0.909±0.033 | 0.928±0.030 | 0.024 |
| DCE_wavelet-LHH_glcm_InverseVariance | 0.447±0.058 | 0.468±0.033 | 0.472±0.031 | 0.070 |
| DCE_log-sigma-6-0-mm-3D_firstorder_Skewness | -0.172±0.374 | -0.288±0.280 | -0.327±0.383 | 0.255 |
| T2WI_wavelet-LLL_gldm_SmallDependenceHighGrayLevelEmphasis | 746.98±850.57 | 396.25±387.72 | 455.58±416.91 | 0.101 |
| T2WI_wavelet-HHH_ngtdm_Complexity | 1.091±1.085 | 0.594±0.501 | 0.546±0.418 | 0.001 |
| T2WI_wavelet-HLH_glcm_DifferenceAverage | 0.425±0.019 | 0.415±0.020 | 0.412±0.017 | 0.007 |
| T2WI_log-sigma-5-0-mm-3D_ngtdm_Complexity | 31.852±36.300 | 15.397±15.416 | 18.388±20.953 | 0.274 |
| T2WI_wavelet-LLL_firstorder_Range | 2276.67±1290.56 | 1901.96±899.95 | 1989.25±909.44 | 0.276 |
| T2WI_log-sigma-2-0-mm-3D_gldm_DependenceEntropy | 6.797±0.890 | 6.724±0.761 | 6.780±0.752 | 0.286 |
| T2WI_wavelet-HHH_glcm_Correlation | 0.053±0.031 | 0.059±0.024 | 0.045±0.026 | 0.033 |
| T2WI_wavelet-HHH_glszm_SmallAreaHighGrayLevelEmphasis | 1.638±1.219 | 1.014±1.062 | 0.925±0.525 | 0.025 |
| T2WI_wavelet-HHL_firstorder_Skewness | 0.028±0.083 | 0.046±0.081 | 0.079±0.066 | 0.016 |
| T2WI_wavelet-HLH_glszm_ZonePercentage | 0.005±0.004 | 0.003±0.002 | 0.003±0.002 | 0.011 |
| T2WI_wavelet-HHH_firstorder_Median | -0.003±0.013 | -0.002±0.011 | 0.002±0.012 | 0.049 |
| T2WI_wavelet-HLH_glcm_Id | 0.789±0.008 | 0.793±0.009 | 0.795±0.008 | 0.009 |
| T2WI_wavelet-HHH_glszm_GrayLevelVariance | 0.335±0.230 | 0.242±0.025 | 0.245±0.036 | 0.144 |
| T2WI_wavelet-LLH_glcm_Imc1 | -0.166±0.019 | -0.177±0.025 | -0.180±0.024 | 0.101 |
| ADC_log-sigma-6-0-mm-3D_glszm_SmallAreaLowGrayLevelEmphasis | 0.005±0.008 | 0.003±0.003 | 0.002±0.002 | 0.339 |
| ADC_log-sigma-3-0-mm-3D_firstorder_Kurtosis | 2.909±0.463 | 3.181±0.653 | 3.350±0.767 | 0.008 |
| ADC_wavelet-LHH_glcm_ClusterShade | -0.017±0.073 | -0.008±0.046 | 0.008±0.055 | 0.253 |
| ADC_log-sigma-4-0-mm-3D_firstorder_Mean | -39.423±44.814 | -51.759±67.825 | -67.556±59.074 | 0.160 |
| ADC_log-sigma-6-0-mm-3D_firstorder_Skewness | -0.094±0.310 | -0.158±0.412 | -0.289±0.419 | 0.077 |
| ADC_wavelet-LHH_gldm_DependenceVariance | 25.802±2.463 | 26.198±2.202 | 26.780±2.027 | 0.064 |
| ADC_wavelet-HHL_glcm_ClusterShade | -0.021±2.125 | -0.551±1.781 | 0.569±3.540 | 0.178 |
| ADC_wavelet-LLH_glcm_Correlation | 0.738±0.041 | 0.729±0.039 | 0.737±0.036 | 0.158 |
| ADC_log-sigma-5-0-mm-3D_glszm_SmallAreaLowGrayLevelEmphasis | 0.004±0.005 | 0.003±0.003 | 0.002±0.001 | 0.431 |

Abbreviations: DCE, dynamic contrast enhancement; T2WI, T2-weighted imaging; ADC, apparent diffusion coefficient; HER2, human epidermal growth factor receptor-2; HR, hormone receptor; TNBC, triple-negative breast cancer; AUC, area under curve.

**Supplementary Figures and legends**

**
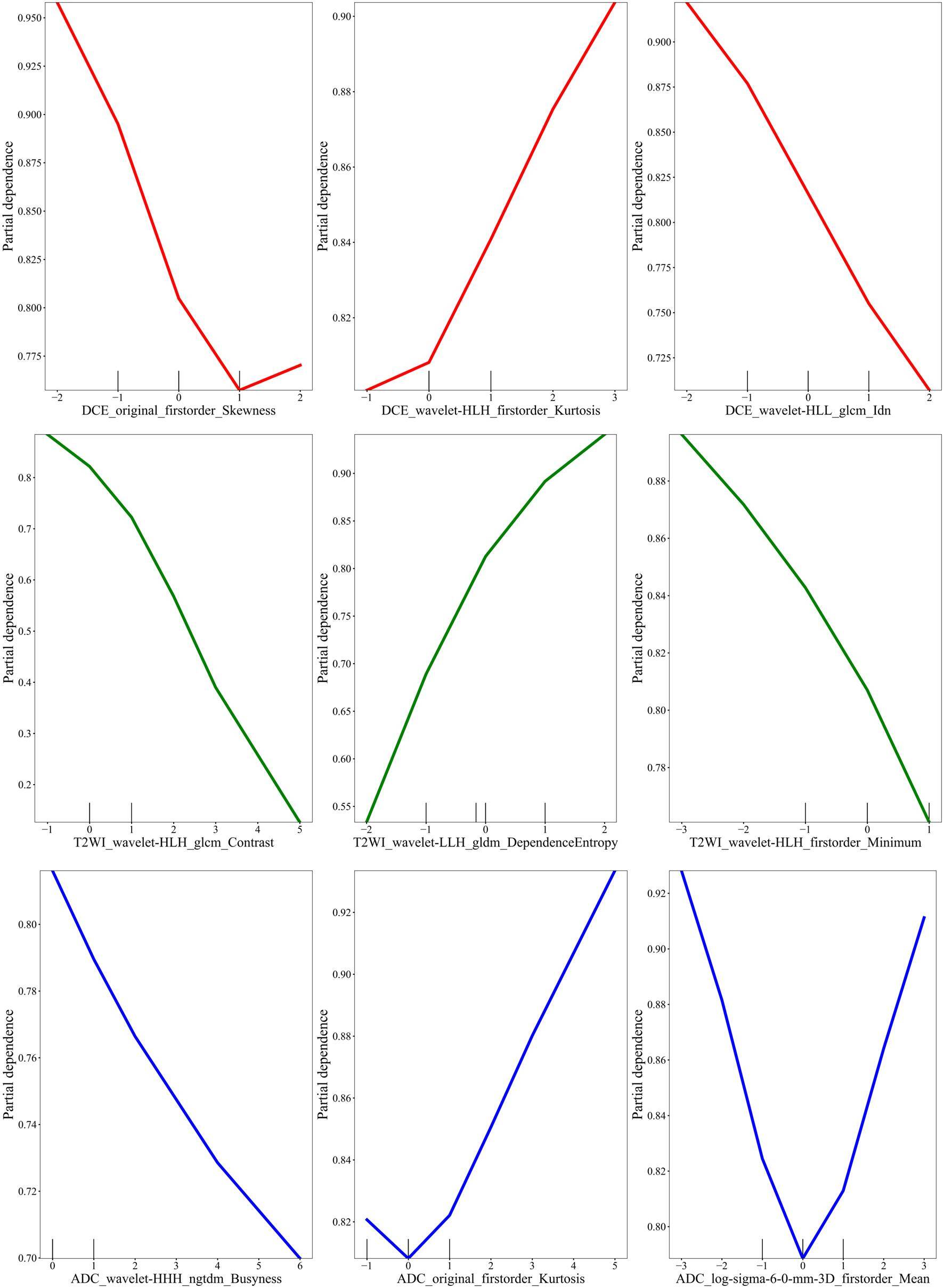
**

**Figure S1.** Partial dependence plot (PDP) of the several representative radiomics features from three MRI sequences correlated to the AR+ expression.


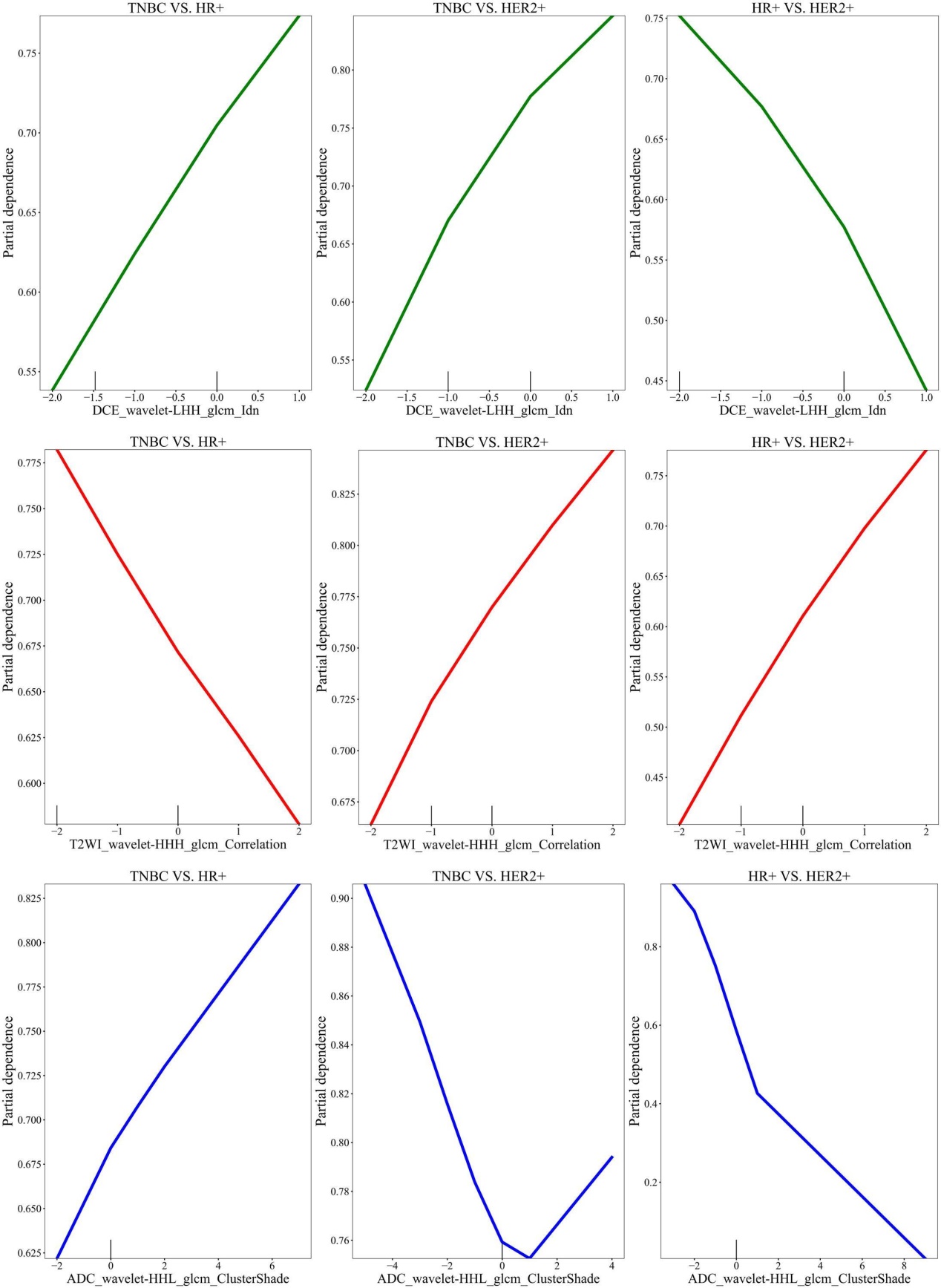


**Figure S2.** Partial dependence plot (PDP) of the several representative radiomics features from three MRI sequences correlated to the molecular subtype.
